# Integrated Immune Profiling Identifies Treatment-Associated Immune Patterns in Patients with B‑Cell Hematological Malignancies

**DOI:** 10.1021/acs.jproteome.6c00394

**Published:** 2026-09-03

**Authors:** Carlota Arias-Hidalgo, Juan Hernández-Vazquez, Cristina Teodosio, Pablo Juanes-Velasco, Ana Nuño-Soriano, Paloma Cal-Sabater, Elisa Arribas-Rodríguez, Aida Fiz-López, Marina Pérez-Mazzali, Álvaro Martín-Muñoz, Pablo Tellería, Marina G. De Vicuña, Javier Gamazo-Herrero, Ignacio de la Fuente-Graciani, Sonia Pérez-González, Sara Bravo, Cristina García-Alonso, Carlos Dueñas, María Jesús Peñarrubia, Manuel Vigo-Aceves, Claudia Núñez-Abascal, Lucía Neira-González, Rafael Gongora, Sara Cuesta-Sancho, David Bernardo, Ángela-Patricia Hernández, Manuel Fuentes

**Affiliations:** † Translational and Clinical Research Program, Cancer Research Center (IBMCC, CSIC-University of Salamanca), Cytometry Service, NUCLEUS, Department of Medicine, University of Salamanca (Universidad de Salamanca), 37008 Salamanca, Spain; ‡ Instituto de Investigación Biomédica de Salamanca(IBSAL), Complejo Asistencial de Salamanca, Paseo de San Vicente, 58, 37008 Salamanca, Spain; § Plataforma de Proteómica Clínica, Instituto de Investigación Biomédica de Salamanca, (IBSAL), 37007 Salamanca, Spain; ∥ Mucosal Immunology Lab, Institute of Biomedicine and Molecular Genetics (IBGM, University of Valladolid-CSIC), 47003 Valladolid, Spain; ⊥ Biomedical Research Networking Centre Consortium of Oncology (CIBERONC), Instituto de Salud Carlos III, 28029 Madrid, Spain; # Flow Cytometry Facility, Institute of Biomedicine and Molecular Genetics (IBGM, University of Valladolid-CSIC), 47003 Valladolid, Spain; ¶ Internal Medicine Department, 16238 Hospital Clínico Universitario de Valladolid, 47003 Valladolid, Spain; ∇ Department of Hematology, Hospital Clínico Universitario de Valladolid, 47003 Valladolid, Spain; ○ Instituto de Investigación Biosanitaria de Valladolid (IBioVall), 47010 Valladolid, Spain; ⧫ Centro de Investigaciones Biomédicas en Red de Enfermedades Infecciosas (CIBERINFEC), 28029 Madrid, Spain; †† Department of Pharmaceutical Sciences, Organic Chemistry, Faculty of Pharmacy, University of Salamanca, CIETUS, IBSAL, 37007 Salamanca, Spain

**Keywords:** oncohematological malignancies, integrated immune profiling, immunocompetence, humoral immune dysfunction, cellular immunophenotyping, multiomics factor analysis, treatment-associated immune signatures

## Abstract

Immune dysfunction in hematological malignancies arises from both disease-related alterations and therapeutic interventions, yet comprehensive approaches for characterizing the immune status remain limited. We evaluated an integrated immune profiling strategy combining antigen-specific serology and high-dimensional B-cell phenotyping. Thirty-eight patients with B-cell hematological malignancies receiving ibrutinib-, lenalidomide-, and rituximab-based therapies, or no treatment, were analyzed. Antigen-specific humoral responses were assessed by using a customized protein microarray containing 37 microbial antigens, enabling parallel IgG and IgM profiling. B-cell subpopulations were characterized by spectral flow cytometry. Multiomics factor analysis was applied to integrate serological and cellular data sets and identify major sources of immune variability. Distinct serological and cellular immune profiles were observed across therapeutic groups. Patients receiving ibrutinib displayed lower IgG levels against several microbial antigens and alterations in multiple B-cell compartments, whereas patients receiving lenalidomide-based therapy showed comparatively preserved B-cell populations and broader antigen-specific IgG reactivity. Rituximab-containing regimens were associated with marked reductions in several B-cell subsets. Integrated analysis identified cellular immune features as the major contributors to interpatient heterogeneity, which suggests the presence of treatment-associated immune patterns within the studied cohort. Therefore, integrated serological and cellular profiling provides a scalable framework for characterizing an immune fingerprint with potential prognostic value in patients with B-cell hematological malignancies. These findings support further investigation of immune monitoring approaches in larger longitudinal cohorts.

## Graphical abstract

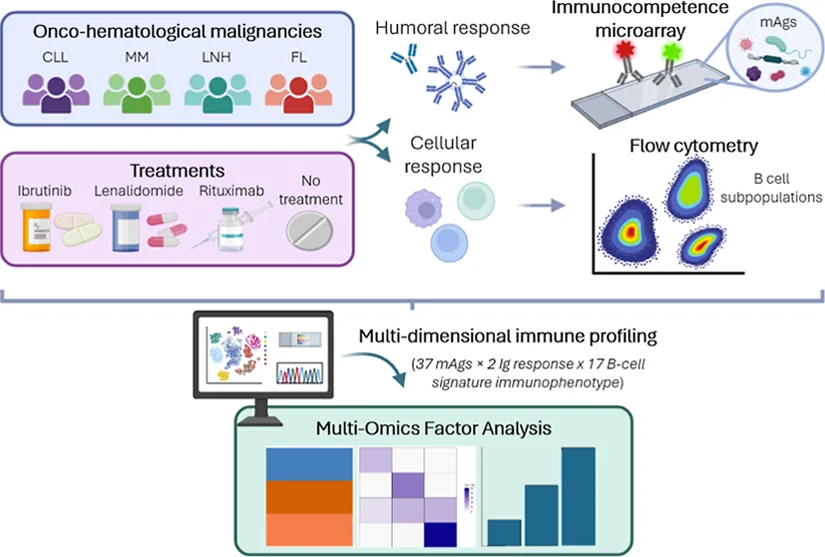

## 1 Introduction

Hematological malignancies comprise a highly heterogeneous group of diseases affecting the blood, bone marrow, and lymphoid organs, which are characterized by widely variable prognoses and a high rate of relapse. They account for 1.2 million new cases worldwide each year, representing about 7% of all newly diagnosed cancers.^1^

Lymphoid malignancies, including lymphomas, leukemias, and multiple myeloma (MM), are characterized by clonal proliferation of immune cells and exhibit substantial biological and clinical heterogeneity. Non-Hodgkin lymphomas (NHLs) arise from mature lymphocytes at different stages of differentiation and encompass indolent to highly aggressive forms, each requiring distinct therapeutic strategies.^2^ Despite major advances in treatment, including targeted therapies and immunotherapies, many patients fail to respond or eventually develop resistance, highlighting an unresolved challenge in disease management.^3^

Follicular lymphoma (FL) is a typically slow-growing, indolent B-cell lymphoproliferative disorder arising from follicular center B-cells. It commonly presents with widespread lymphadenopathy, bone marrow involvement, and splenomegaly and less commonly other extranodal sites. FL is the second most common type of NHL, representing about 30%.^4,5^ Leukemias originate from hematopoietic cells in the bone marrow and lead to the accumulation of abnormal blood cells.^1^

Chronic lymphocytic leukemia (CLL), one of the most common leukemias, is defined by the expansion of CD5+ B-cells and profound immune dysregulation. Malignant B-cells in CLL show resistance to apoptosis and impaired immune activity, resulting in both humoral and cellular immune dysfunction.^6,7^ Additionally, CLL is also associated with significantly altered T-cell function, which compromises antitumor immunity and increases susceptibility to infections.^8,9^ Currently, several therapeutic options are available for leukemias and lymphomas, such as chemotherapy, monoclonal antibodies (i.e., rituximab), kinase inhibitors (i.e., ibrutinib), and advanced cellular therapies such as CAR-T cells.^10,11^ Although these therapeutic approaches have substantially improved patient outcomes, therapeutic resistance remains a major limitation for these pathologies.

MM is characterized by clonal expansion of plasma cells in the bone marrow and has benefited from treatments such as proteasome inhibitors, immunomodulatory drugs like lenalidomide, monoclonal antibodies, and autologous stem cell transplantation. Nevertheless, the disease remains largely incurable, particularly in advanced stages.^12^ Increasing evidence indicates that MM therapies are associated with significant immune alterations, leading to an increased risk of infections, including bacterial, viral, and opportunistic infections and reactivation of latent pathogens.^12,13^

Infections represent a major complication in these onco-hematologic malignancies due to disease-related immune dysregulation and treatment-induced immunosuppression.^14^ In CLL patients, viral infections may act as a triggering factor in the early stages of the disease, potentially promoting clonal expansion through chronic antigenic stimulation.^15^ Patients with MM, NHL, and FL also show increased susceptibility to infections due to impaired antibody production and T-cell dysfunction, further exacerbated by therapies such as chemotherapy and targeted agents.^2,16,17^ The infection severity and resolution (with or without sequelae) are influenced by the immune resilience and immunocompetence of the patient,^18^ which encompasses mechanisms that restore immune function and maintain self-tolerance.^19,20^ Immunological memory generated by previous infection or vaccination can mitigate reinfections and reduce severity.^21–23^ Humoral immunity, mediated by antibodies produced by B-cells, is crucial. B-cells constitute a heterogeneous population of functionally distinct phenotypic subsets well-defined by their maturation status and immunophenotype expression, each contributing differently to antibody production, immune memory, and pathogen neutralization.^24–27^ In these pathologies, functional B-cells are often depleted or replaced by malignant clones, reducing the ability to sustain effective responses.^26,28^ Consequently, immune protection and vaccine-induced immunity are highly variable among patients and are influenced by disease stage, tumor burden, and immune impairment.^28,29^

Currently, the relative impact of several antitumor therapies on humoral and cellular immune responses, particularly in these oncohematological diseases, remains poorly defined and not well understood.^30^ Although targeted therapies are designed to reduce secondary effects, these patients often present intrinsic immune dysfunctions.^31,32^ Immunosuppression is observed with the initiation of the treatment and disease progression due to cumulative therapeutic exposure.^31,33^ Therefore, early prognosis might be crucial to enable risk-adapted treatment strategies and improve the prevention of infections.^34^

Our aim is to characterize antigen-specific humoral responses of patients with oncohematological malignancies undergoing different therapeutic regimens (containing ibrutinib, lenalidomide, or rituximab). For this purpose, serological profiling using a functional antigen microarray was employed to assess their immune response against 37 common microbial antigens (mAgs), and B-cell immunophenotyping was carried out by spectral flow cytometry. By integrating these data sets, we sought to determine how patients receiving different therapeutic regimens differ in humoral and cellular immune profiles, revealing discordance that ultimately shapes pathogen recognition, host defense, disease evolution, and postinfection sequelae. This comprehensive analysis provides insights into immune signatures associated with vaccine responsiveness, recurrent infections, and susceptibility to common pathogens in the population.

## 2 Materials

Ethanol 96%, 3-(2-aminoethylamino)-propyldimethoxymethylsilane (MANAE) (≥95.0%), bis­(sulfosuccinimidyl)­suberate (BS3), glycerol 85%, bovine serum albumin (BSA), fetal bovine serum (FBS), Tween 20, dimethyl sulfoxide (DMSO), Hybriwell sealing system, Grace Bio-Laboratories ProPlate microarray system, Grace Bio-Laboratories ProPlate clips for microarray systems (Sigma-Aldrich, St. Louis/MO, USA), SuperBlock Tris-buffered saline (#37535), Pierce bicinchoninic acid Protein Assay Kit (#23225), Blocker BSA (10%) in phosphate buffered saline (PBS) (#37525), microscope slides (76 × 26 mm), Mseries Lifterlip y Lifterslip coverslips, Live/Dead Fixable Blue Dead Cell Stain Kit, Gibco RPMI 1640 Medium (Thermo Scientific, Rockford/IL, USA), Microarray-Specific 384-well Microplates, Optimum Microarray Printing Buffer C (ArrayJet, Roslin, UK), Streptavidin-Fluor Cy3 Cytiva Amersham, Cytiva Ficoll–Paque PLUS (Cytiva 17–1440–03) (GE-Healthcare, Little Chalfont, Buckinghamshire, UK), Cy3-AffiniPure Donkey Anti-Human IgG (cat. no. 709–165–149), Alexa Fluor 647-AffiniPure Goat Anti-Human IgM (cat. no. 109–605–043) (Jackson ImmunoResearch, Ely, UK), Brilliant Stain Buffer (#563794) (BD Biosciences, San Jose, CA), and True-Stain Monocyte Blocker (#426101) (BioLegend, San Diego, CA) were used. The mAgs included in the array are listed in Table S1, and the antibodies used for B-cell immunophenotyping are listed in Table S2.

### 2.1 Equipment and Software

ArrayJet Printer Marathon v1.4, JetSpyder 12 samples, JetStar (ArrayJet, Roslin, UK), Scanner SensoSpot Fluorescence (Miltenyi Imaging GmbH, Radolfzell, Germany), GenePix 4000B Microarray Scanner, GenePix Pro Microarray Analysis Software (Axon, Molecular Devices, San Jose, CA, USA), Orbital shaker (FALC Instruments S.r.l.; Treviglio, Italy), Fisherbrand Microplate Vortex Mixers (Fisherbrand, EEUU), Digital Dry Block Heater (Thermo Scientific, Rockford/IL, USA), 5-laser Aurora spectral cytometer (Cytek Biosciences, CA, USA), R statistics software (R Foundation for Statistical Computing, Vienna, Austria. http://www.R-project.org/), Infinicyt software (version 2.1.0.a.000; BD Biosciences, San Jose, CA) were used.

## 3 Experimental Procedures

### 3.1 Clinical Characteristics of the Studied Cohort

An oncohematological cohort of 38 patients (Coheńs *d* ≥ 0.5), aged 51 to 76 years, has been studied. Plasma was collected in Hospital Rio Hortega (Valladolid, Spain) and processed after written informed consent of each patient was obtained. The clinical information on the patients is summarized in Table 1, and further clinical information is collected in Table S3.

**Table 1:** Clinical Characteristics of the Patients^e^

disease	treatment	sex
CLL^a^ (*n* = 20, 53%)	ibrutinib (*n* = 15, 75%)	female (*n* = 10, 50%)
	no treatment (*n* = 5, *25%)*	male (*n* = 10, 50%)
MM^b^ (*n* = 8, 21%)	lenalidomide-based (*n* = 8, *100%)*	female (*n* = 5, 62.5%)
		male (*n* = 3, 37.5%)
NHL^c^ (*n* = 8, 21%)	R–CHOP (*n* = 8, 100%)	female (*n* = 2, 25%)
		male (*n* = 6, 75%)
FL^d^ (*n* = 2, 5%)	no treatment (*n* = 2, 100%)	female (*n* = 2, 100%)

a Abbreviations: CLL: chronic lymphocytic leukemia.

b MM: multiple myeloma.

c NHL: non-Hodgkin lymphoma.

d FL: follicular lymphoma.

e R–CHOP: R—Rituximab, C—Ciclofosfamida, H—Hidroxidaunorubicina, O—Oncovin, P—Prednisona.

### 3.2 Profiling Antimicrobial Antibodies

#### 3.2.1 Immunocompetence Microarray (mAg Array) Design

The mAg microarray was designed to include 37 mAgs together with appropriate positive and negative controls.^35^ Detailed information is provided in Table S1.

The microarray was printed with 7 identical subarrays with 684 spots. Each subarray contained 5 dilutions of the mAgs (range from 8 × 10^–2^ to 1 × 10^–5^ μg/μL) in PBS 1× Na^+^/K^+^. All the antigens were printed in triplicate. Positive (Cy3, anti-IgG antibody, Cy3 anti-IgG, AlexaFluor647 anti-IgM) and negative (Blocking Buffer, PBS, PBS-Tween, Printing buffer, and Clean buffer) controls were also included in each subarray. The microarray content was deposited on a previously chemically activated glass surface, prepared according to previous reports,^36^ via a noncontact printer, and diluted 1:1 (v/v) in Arrayjet printing buffer C, following ArrayJet Printer Marathon v1.4 specifications. Printed arrays were stored protected from light at room temperature (RT) for 2 days and at 37 °C for 10 days. They were packed and stored at RT until they were assayed.

#### 3.2.2 Serological Screening by mAg Array

Following previously described protocols,^35,37^ microarrays were blocked with Blocker BSA 1× for 1 h with slight shaking and washed (3×, 5 min) with distilled water. Plasma samples were diluted 1:10 (v/v) in Blocker BSA 1× and incubated overnight at 4 °C in an orbital shaker. The following day, microarrays were washed (3×, 5 min) with PBS-Tween 0.05% at RT with mild stirring. A mix of secondary antibodies (Cy3-AffiniPure Donkey Anti-Human IgG and Alexa Fluor 647-AffiniPure Goat Anti-Human IgM) was prepared at a final dilution of 1:1000 (v/v) in PBS-Tween 0.05%. The incubation was conducted with 200 μL/microarray for 1 h at RT. Finally, microarrays were washed with PBS-Tween 0.05% (3×, 5 min), dried, and scanned.

#### 3.2.3 Image Acquisition

The scanning was performed with a GenePix 4000B Microarray Scanner. The parameters were set to quantify signal-intensity values at Cy3 (λ = 532 nm) and Alexa Fluor 647 (λ = 635 nm). The TIFF images generated were analyzed using GenePix Pro 6.0 software.

### 3.3 Isolation, Storage, and Staining of Peripheral Blood Mononuclear Cells for Flow Cytometry Analysis

In all cases, peripheral blood was collected in tubes containing lithium heparin as anticoagulant. Prior to isolation of peripheral blood mononuclear cells (PBMCs), plasma was collected and stored at −80 °C. Isolation of PBMCs was performed using Cytiva Ficoll–Paque PLUS following the manufacturer’s instructions.

Peripheral blood was added to Ficoll–Paque without mixing at a ratio of Blood/Ficoll of 5:3 (v/v). Samples were centrifuged at 800 g for 30 min at 4 °C with acceleration set to maximum and deceleration to minimum. PBMCs were collected and washed with RPMI 1640 medium at 400 g for 5 min at 4 °C. The resulting pellet was frozen for posterior analysis. Freezing was performed in freezing medium containing 10% DMSO diluted in FBS. The sample was then frozen using a temperature gradient and stored in liquid nitrogen before thawing and further processing. Sample staining was performed employing 2 × 10^6^ thawed PBMCs and a modified version of the OMIP-069 protocol and analysis panel, optimized for spectral flow cytometry.^38^ Briefly, prior to antibody staining the PBMCs, the Live/Dead Fixable Blue Dead Cell Stain Kit was added to exclude dead cells from the analysis. Brilliant Stain Buffer and True-Stain Monocyte Blocker were also added prior to staining with the antibodies to obtain optimal fluorescence of the desired cells. The PBMCs were washed with fluorescence-activated cell sorting (FACS) buffer (500 mL PBS + 10 mL filtered FBS + 0.1 g NaN3 + 2.5 mL sterile ethylenediaminetetraacetic acid) and incubated in the dark at RT during the staining process. Finally, the cells were fixed with 0.8% paraformaldehyde in FACS buffer in the dark for 10 min, washed with FACS buffer, and stored at 4 °C.

### 3.4 Flow Cytometry Gating Strategy

Cells were acquired within 48 h in a 5-laser Aurora spectral cytometer. Data analysis was performed using Infinicyt software. The complete gating strategy for all analyzed immune populations, including representative plots for each sequential gating step, is provided in Figure S1.

The gating strategy was designed to accurately identify major leukocyte and lymphocyte populations from the peripheral blood samples. First, a time gate was applied to ensure acquisition stability and exclude potential artifacts related to signal fluctuations during data collection. PBMCs were then identified on the basis of forward scatter (FSC) and side scatter (SSC) properties, allowing the exclusion of debris. Subsequently, singlet cells were selected by using FSC-A versus FSC-H parameters to eliminate doublets and cell aggregates. Cell viability was assessed using a live/dead discrimination dye, and only viable cells were included in the downstream analyses. Leukocytes were then identified on the basis of CD45 expression. B-cells were identified by CD19 expression, while non-B-cells (CD19^–^) were excluded. In parallel, T cells were defined as CD3^+^ lymphocytes. Within the B-cell compartment, memory B-cells (MBCs) were sequentially identified according to IgD and CD27 expression. Double-negative (DN; IgD^–^CD27^–^) MBC were further classified into DN1 (CD11c^–^CXCR5^+^), DN2 (CD11c^+^CXCR5^–^), and DN3 (CD11c^–^CXCR5^–^), using the combined expression of CD11c, CXCR5, CD21, and FcRL5.

This sequential gating strategy ensured robust and reproducible identification of both major and rare immune-cell subsets. Rare populations were quantified only when a minimum number of events were detected. The B-cell subpopulations included in the spectral flow cytometry analysis are given in Table 2. Comprehensive results, including total event counts, their frequencies, and the corresponding FSC/SSC characteristics for each subset, are reported in Table S4.

**Table 2:** B-Cell Subpopulations Analyzed by Spectral Flow Cytometry

main population	subpopulation	signature immunophenotype
Pre-GC^a^	immature	CD24bright CD38bright (T1^d^)
		CD24+ CD38+ (T2)
		CD24dim CD38dim (T3)
	naive	CD24+ _IgMlo/IgM+
		CD24– CD38– CD95+ CD11c+
		CD24– CD95–_IgMlo/IgM+
MBC^b^	nonswitched	CXCR5+
		CXCR5– CD95+
	switched	CD27– CD21+ CXCR5+ CD11c– (DN1^e^)
		CD27– CD21– CXCR5– CD11c+ (DN2)
		CD27– CD21– CXCR5– CD11c– (DN3)
		CD27+ CXCR5+
		CD27+ CXCR5– CD95+ CD11c-
PB^c^	IgM+	CD20+ (early PB)
		CD20– (late PB)
	IgM–	CD20+ (early PB)
		CD20– (late PB)

a Abbreviations: Pre-GC: pregerminal center B-cells.

b MBC: memory B-cells.

c PB: plasmablasts.

d T: transitional B-cells.

e DN: double-negative B-cells.

### 3.5 Data Analysis and Bioinformatics

#### 3.5.1 Data Preprocessing and Quality Control

The fluorescence signals obtained from the images were adjusted by subtracting the background signal and subsequently converted into *Z*-scores, following the method outlined in previous studies.^39,40^ To verify the accuracy of signal correction for each microarray, both the raw fluorescence data and the log2 density distribution (*Z*-score) were compared (Figure S2). Principal component analysis (PCA) was conducted to discard any potential batch effect associated with the microarray (Figure S3). All data analyses were carried out using the R environment.

#### 3.5.2 Biostatistics and Data Visualization

Boxplots and volcano plots display the statistically significant differences in the *Z*-score ratio between two specific conditions. The *Z*-ratio is computed by subtracting the mean *Z*-score in each condition and dividing by the overall standard deviation of *Z*-score in both conditions, as previously described.^41^ In the volcano plot, the *Y*-axis indicates the statistical significance of the mean *Z*-score difference between conditions A and B, calculated using the Wilcoxon rank-sum test, with values expressed as −log2­(*p*-value).

All dendrograms shown in the heatmaps were created by clustering within the group means. Clustering is first performed within each group, followed by clustering of the group means, and the within-group and between-group dendrograms are linked using the merge_dendrogram function in R. The plots presented in this study were generated using the following R packages: ggplot2, ggpubr, grid, gridExtra, circlize, ggrepel, ggsignif, and ComplexHeatmap.

The integration of the different data sets was performed using a Multi-Omics Factor Analysis (MOFA), an unsupervised framework implemented in RStudio with the MOFA2 package.^42^ Prior to model training, each data set was normalized independently to ensure comparability across data modalities. Factors explaining ≥1.5% of the variance in at least one omics data set were retained for downstream analyses,^43^ excluding factors with negligible variability while preserving biologically meaningful sources of variation.

To evaluate the performance of the classifier, receiver operating characteristic (ROC) curves were generated by plotting the true positive rate against the false positive rate at different classification thresholds. Model performance was assessed using accuracy, sensitivity, specificity, precision, F1-score, and the area under the curve (AUC). To reduce overfitting and estimate the model’s generalization performance, a k-fold cross-validation strategy was applied during model training. Feature selection was performed within each training fold to identify the most informative variables and reduce model complexity. The AUC was calculated to quantify the model’s discriminative ability.

## 4 Results

### 4.1 Humoral Immune Profile in Oncohematological Patients

To characterize antigen-specific antibody profiles across therapeutic groups, an exhaustive characterization of the humoral profile was performed. While a distinct serological profile is observed among the different groups (Figure 1), PCA revealed that the heterogeneity in mAg profiles was not fully explained only by the treatment (Figure S4). High variations in IgG levels have been observed in the analysis of the different therapies (Figures 1 and S5). Figure 1A shows that there was a distinct serological profile between treated vs nontreated individuals. Lower levels of IgG against mAgs from *Cytomegalovirus*, measles virus, SARS-CoV-2, and varicella-zoster were consistently observed in treated patients, regardless of the administered treatment (Figure 1B). In addition, the volcano plots revealed differences across each treatment modality in the IgG profile (Figure 1C). Among the key findings, levels of IgG antibody against Norovirus, *Streptococcus pneumoniae*, and SARS-CoV-2 antigens were significantly elevated in lenalidomide- or rituximab-based treatments, compared with ibrutinib (Figure 1D). Interestingly, the IgM serological profile did not differ among the distinct groups, except for the *S. pneumoniae* antigen (Figures S6 and S7). Levels of this IgM antibody were higher in patients treated with rituximab, which may reflect a distinct effect on early immune responses to bacterial infections in comparison with other therapies.

**Figure 1:**
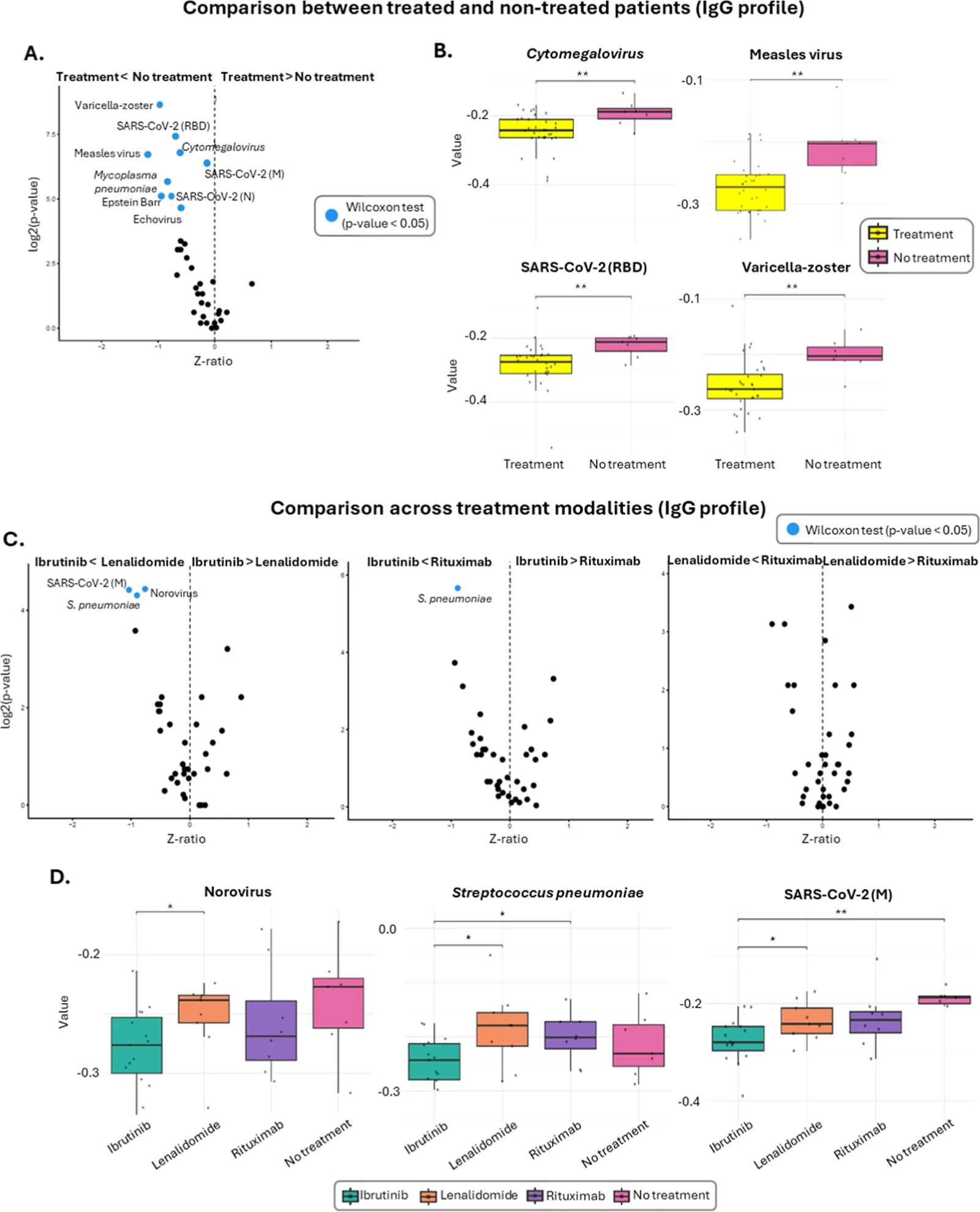
Effect of different treatment modalities on the IgG profiles. (A) Volcano plot displaying the statistical significance of *Z*-score ratios (*X*-axis) between treated and nontreated patients. Statistical differences between groups were assessed using the Wilcoxon rank-sum test (*Y*-axis). The mAgs with statistically significant differences are highlighted in blue dots. (B) Boxplots of the IgG humoral profile comparing treated and nontreated patients. Boxplots show the median (horizontal line) and interquartile range (box). Yellow represents patients with treatment, and pink represents those with no treatment. Statistical differences between groups were assessed using the Wilcoxon rank-sum test (*p*-value: * <0.05; ** <0.01). (C) Volcano plots highlighting the statistical significance of *Z*-score ratios between ibrutinib, lenalidomide, and rituximab. Each volcano plot represents the comparison between treatment modalities. (D) Boxplots of the IgG humoral profile against mAgs according to treatment. Each color represents a different group: green for ibrutinib, orange for lenalidomide, purple for rituximab, and pink for no treatment.

Together, these findings revealed treatment-associated differences in serological profiles against common mAgs and may be useful for immune monitoring. Moreover, differential serological profiles might represent potential biomarkers because of their ability to discriminate between patient groups.

Among CLL patients, those who received ibrutinib exhibited lower IgG levels against several pathogens (such as cytomegalovirus, *Mycoplasma pneumoniae*, SARS-CoV-2 (N), and varicella-zoster) (Figures 2 and S8). While these findings suggested a treatment-associated impairment of humoral immunity (potentially affecting vaccine-induced immunity), clinical factors, such as age and sex, may also contribute to the observed variability in immune responses. The reduced IgG levels observed in ibrutinib-treated patients may, therefore, reflect a combined effect of therapy and host-related factors, suggesting reduced serological responses against previously encountered pathogens. This highlights the importance of monitoring potential reactivation of infections or increased susceptibility to new ones, given their compromised immune status due to the impairment of the immune response. These findings suggest that monitoring humoral immunity during treatments containing ibrutinib may facilitate infection risk assessment and support preventive clinical strategies. Therefore, a thorough analysis of the humoral profile may contribute to immune-monitoring strategies in CLL.

**Figure 2:**
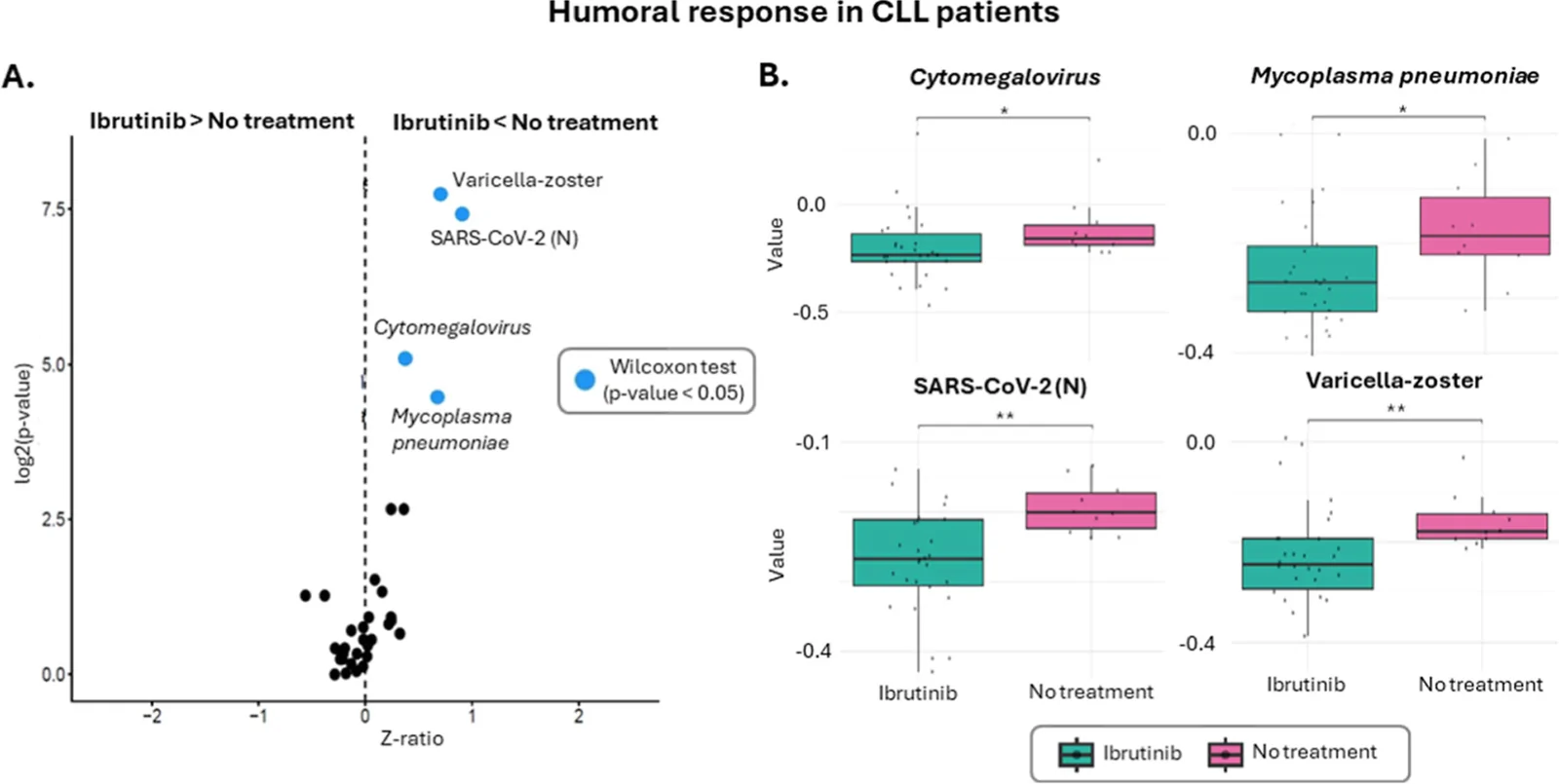
Comparison of the humoral profile in Ibrutinib-treated patients with CLL. (A) Volcano plot displaying the statistical significance of *Z*-score ratios (*X*-axis). Statistical differences between groups were assessed using the Wilcoxon rank-sum test (*Y*-axis). The mAgs with statistically significant differences are highlighted in blue dots. (B) Boxplots of the humoral profile against mAgs. Boxplots show the median (horizontal line) and interquartile range (box). Statistical differences between groups were assessed using the Wilcoxon rank-sum test (*p*-value: * <0.05; ** <0.01).

### 4.2 Treatment-Associated Differences on B-Cell Subpopulations

Since all patients in this study cohort had B-cell malignancies, multiple B-cell subpopulations were evaluated to contextualize immune alterations and determine their relationship with humoral responses.

Differences in B-cell subpopulation profiles across treatment groups were explored by PCA. The biplot revealed a clear separation of individuals according to treatment, indicating distinct immunological signatures. MBCs contributed mainly to positive values of both dimensions, whereas plasmablasts (PBs) were associated with negative values in the second dimension. Pregerminal center (pre-GC) B-cells were key drivers of sample dispersion. Overall, these results suggested that treatment regimens were linked with specific shifts in B-cell differentiation states (Figure S9).

Overall, the coordinated reduction of pre-GC B-cells, MBC, and PB reflected reduced frequencies compatible with altered B-cell differentiation in all the groups (Figure 3). Figure 3A shows that both ibrutinib- and rituximab-containing regimens reduced the number of B-cells in multiple subsets regardless of their function and differentiation. Treatments including rituximab were associated with the lowest frequencies, from early B-cell differentiation stages to antibody-secreting cells, while those including ibrutinib showed an intermediate effect mainly impacting pre-GC B-cells and PB. Therefore, while rituximab may induce broad B-cell depletion, suggesting impaired humoral immunity, ibrutinib primarily interfered with early B-cell activation and germinal center formation, without complete cellular ablation/depletion. In contrast, treatments containing lenalidomide seemed to generally preserve the B-cell compartment, with maintained MBC and PB frequencies, but increased pre-GC B-cells. These findings highlighted distinct immunological impacts of each studied therapy, underscoring the need to balance therapeutic efficacy with preservation of immune status, particularly in the context of long-term humoral immunity and the risk of infection.

**Figure 3:**
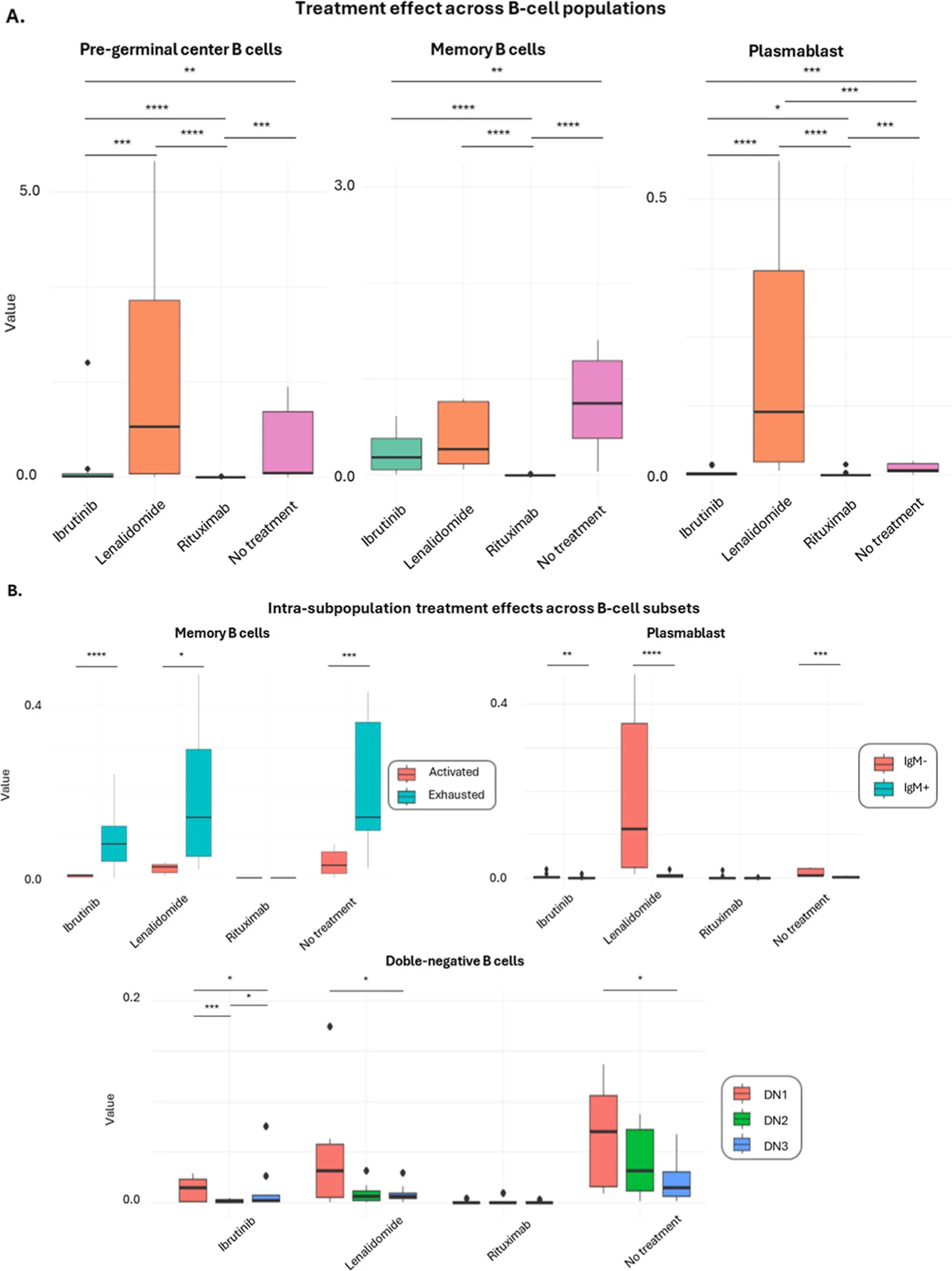
(A) Distribution of B-cell populations across patient groups stratified by treatment. (B) Boxplots depicting treatment-associated differences within specific B-cell subpopulations (activated/exhausted MBCs, IgM^+^/IgM^–^ plasmablasts, and double-negative MBCs). Boxplots show the median (horizontal line) and interquartile range (box). Statistical differences between groups were assessed using the Wilcoxon rank-sum test (*p*-value: * <0.05; ** <0.01; *** <0.001; **** <0.0001).

The MBC compartment was assessed by comparing activated (CD27^+^ CXCR5^–^ CD95^+^ CD11c^+^) and exhausted (CD27^+^ CXCR5^+^) populations (Figure 3B). Across patients treated with ibrutinib, with lenalidomide, and nontreated individuals, the exhausted subset consistently outnumbered the activated subset, suggesting an altered distribution of MBC subsets. Further analysis of the MBC compartment was focused on double-negative (DN) B-cell subsets (DN1, DN2, and DN3). Among these, the most notable differences were observed in DN1. Commonly, DN1 cells are associated with resting or classical memory phenotypes, whereas DN2 and DN3 subsets are linked to increased activation and pro-inflammatory profiles. The relative alteration of DN1 frequencies may indicate a shift in MBC homeostasis, potentially reflecting impaired maintenance or altered differentiation, which can ultimately affect the long-term immunocompetence and responses to subsequent infections. Finally, analysis of the PB compartment revealed a predominance of IgM^–^ over IgM^+^ PB in ibrutinib- and lenalidomide-based regimens and nontreated individuals, indicating a bias toward class-switched effector responses across groups.

The integration of cellular immunophenotyping data and serological profiles via correlation analysis revealed a predominant involvement of IgM-associated responses with distinct patterns across several cellular compartments. PB displayed widespread correlations with the serological profile against several antigens corresponding to viral pathogens, particularly within the IgM isotype, indicating strong associations with recent or ongoing immune activation. MBC compartments showed some statistically significant correlations in both responses (IgM and IgG), underscoring a consistent pathogen-linked memory response. Pre-GC B-cells exhibited lower significant associations across Ig isotypes and analyzed mAgs. Importantly, the serological profiles, with negative (red) and positive correlations (blue), revealed pathogen-specific directional effects, with IgM responses suggesting coordinated expansion or activation of these populations in response to pathogen exposure (Figure S10).

### 4.3 Analysis of Treatment-Associated Impairment of Immune Responses

A comprehensive characterization of immune alterations requires an integrated evaluation of both antigen-specific immunoglobulin responses and B-cell immunophenotypes. To address this complexity, in this cohort of patients, the humoral and cellular immune compartments were profiled using multipronged proteomics-based approaches and immune phenotyping by spectral flow cytometry. These data sets, capturing distinct functional aspects of immune activity, were subsequently integrated through multiomics factor analysis (MOFA), enabling the identification of shared and modality-specific sources of variation across this cohort of patients.

MOFA identified four latent factors (LFs), each of which accounted for ≥1.5% of the variance in at least one data set (Figure 4A). Among the data sets, the cellular response contributed the largest share of explained variability. Notably, the immunophenotype analysis of the cellular compartment (B-cell subpopulations) emerged as the dominant source of biological heterogeneity, explaining over 60% of the cumulative variance. In comparison, the serological profiles (IgM and IgG) accounted for 40% and 17% of the variance, respectively. These results suggested that B-cell subpopulations—more than the presence of antigen-specific antibodies—were the main drivers of heterogeneity in the integrated data. To identify the key contributors within each data set, we selected the top 10 analytes with the highest weights for each LF (Figure 4B). The first two LFs (#1 & #2) were predominantly driven by the cellular response, mainly reflecting pre-GC B-cell populations. In contrast, LFs #3 and #4 were largely explained by the serological profile, independent of antigen origin (viral, bacterial, or fungal) (Figure S11).

**Figure 4:**
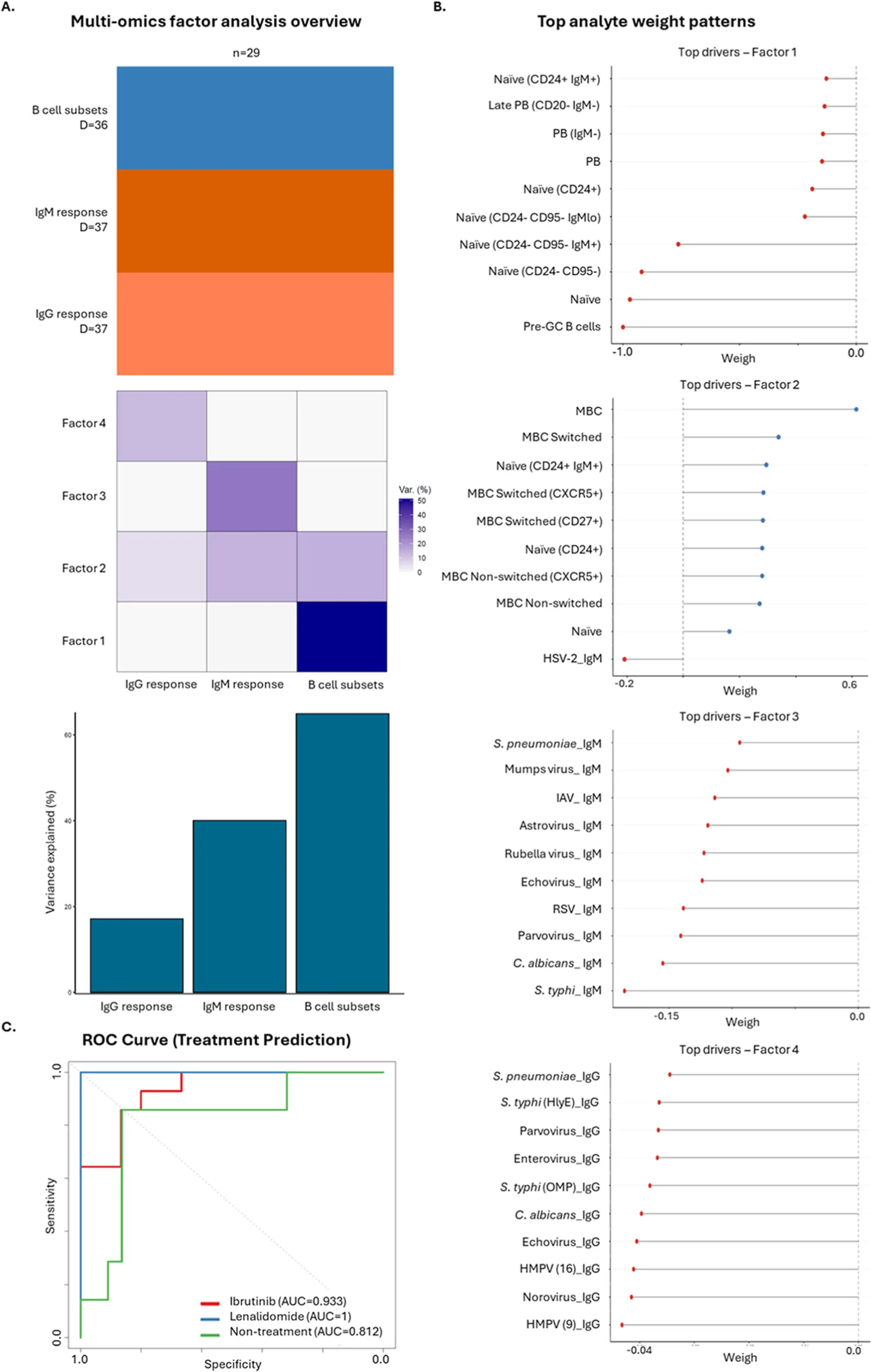
MOFA of immune response in oncohematological patients. (A) The upper panel summarizes the study design, with rows representing modalities (*D*, number of features) and columns indicating patients (*n*). The middle panel shows the variance explained by each LF per data set, while the lower panel depicts the cumulative variance. (B) Heatmaps of the top 10 analyte weights for each LF. Abbreviations: Pre-GC, pregerminal center; MBC, MBCs; PB, plasmablasts; HSV-2, Herpes simplex virus type 2; *S. pneumoniae*, *Streptococcus pneumoniae*; IAV, Influenza A virus; RSV, respiratory syncytial virus; *C. albicans*, *Candida albicans*; *S. typhi*, *Salmonella typhi*; HMPV, human metapneumovirus. (C) ROC curves for treatment-based prediction. ROC analysis shows the discriminative performance of three therapeutic groups for predicting the clinical outcome in the studied cohort.

To explore the potential clinical applicability of these integrated immune signatures for patient stratification, ROC analysis was performed across three groups of patients defined by therapeutic regimen (containing ibrutinib, lenalidomide, or no treatment) (Figure 4C). The group treated with lenalidomide displayed clear discrimination, with an AUC of 1.00, indicating optimal classification within the studied cohort. The group treated with ibrutinib also demonstrated strong predictive performance (AUC = 0.933). In contrast, the nontreated group showed more limited discriminative capacity (AUC = 0.812), indicating moderate accuracy. Although this value still reflected acceptable classification performance, it suggested that the balance between sensitivity and specificity may be less robust compared with therapeutic interventions.

Taken together, these ROC curves highlighted substantial differences in the predictive value among the treatment modalities, with lenalidomide showing the greatest predictive value. This may reflect treatment-related immunological and clinical features in oncohematological patients. To assess the robustness of the ROC curve evaluating response to lenalidomide-based therapies, we performed a permutation test based on 100 random shuffles. The distribution of AUC values obtained under the null hypothesis of no association ranged from 0.40 to 0.90, with a symmetric peak around 0.65 (Figure S12). In contrast, the real model yielded an AUC of 1.0, as represented by a red dashed line in the histogram. This value lay entirely outside the range of permuted AUCs, indicating that it was not due to random variability. Although the classifier achieved high performance within the analyzed cohort, the limited sample size and lack of external validation precluded definitive conclusions regarding generalizability.

## 5 Discussion

Our study provides an integrated characterization of serological and B-cell immune profiles in patients with B-cell hematological malignancies receiving different therapeutic regimens, including ibrutinib-, lenalidomide-, and rituximab-containing treatments. Distinct serological and cellular immune patterns were observed across the therapeutic groups, highlighting the heterogeneity of immune alterations and immune status of these patients. Since both the underlying disease and treatment can influence humoral and cellular immunity (particularly B-cell subpopulations and antigen-specific antibody responses), a comprehensive assessment of immune status is revealed by an integration of serological profiling with high-dimensional B-cell immunophenotyping.

As might be expected, distinct patient subgroups exhibited heterogeneous serological profiles defined by the presence of IgM and IgG antibodies against common mAgs. These serological profiles offer important insights into how serological immunity markers and immune dysregulation may influence disease evolution and infection susceptibility. Understanding pathogen-specific antibody profiles in individuals with reported previous infections may provide valuable information for guiding clinical management of these patients. This has been considered particularly important in infections such as SARS-CoV-2, as it may influence the course of the infection, vaccine efficacy, and hospitalizations.^44^ Additionally, it has been demonstrated that some individuals are more prone to reinfection, highlighting the importance of understanding the impact of prior infections and long-lasting antibody responses.^45^

Our results reveal how the B-cell compartment is differentially reshaped beyond the antitumoral treatments (ibrutinib-, lenalidomide-, or rituximab-based therapies), because of distinct observed effects on B-cell differentiation, activation, and effector function. Patients receiving lenalidomide-based therapies displayed comparatively higher frequencies of pre–GC B-cells, MBC, and PB subpopulations, together with stronger IgG responses against viruses primarily affecting the respiratory and/or gastrointestinal tracts. These findings are consistent with the immunomodulatory role of lenalidomide, resulting in more effective antigen-dependent B-cell differentiation and comparatively preserved humoral responses. In contrast, Rituximab-based therapies were associated with profound remodeling of the B-cell compartment due to depletion of CD20^+^ B-cells. Despite this, these patients retained a relatively strong capacity to mount immune responses against multiple mAgs and exhibited a selective increase in IgM reactivity to *S. pneumoniae*, consistent with the known increased infection risk reported in the literature caused by the depletion of B-cells and the associated impaired humoral immunity.^17,46–50^ These observations underscore the importance of detailed infection surveillance and tailored preventive strategies in the population undergoing therapy.

Ibrutinib-treated patients showed lower IgG responses against several mAgs (including Norovirus, *S. pneumoniae*, SARS-CoV-2, and Varicella-Zoster virus) together with reduced frequencies of pre-GC B-cells, MBCs, and PBs, suggesting altered B-cell activation and differentiation. These findings are consistent with the known effects of Bruton’s tyrosine kinase inhibition on B-cell receptor signaling and humoral immune responses,^31,51–59^ as well as the increased risk for opportunistic infections.^60,61^ In addition, changes in memory B-cell subsets, including reduced frequencies of DN1 (associated with long-lived immune protection) and a relative increase in pro-inflammatory DN2/DN3 populations, may reflect alterations in B-cell homeostasis previously associated with inflammatory and activated immune states.^62–64^ Collectively, these observations suggest that treatments containing ibrutinib compromise both antibody production and long-term immunity, which may contribute to impaired humoral responses to infections and vaccination.^65–69^

Overall, the data showed that ibrutinib- and rituximab-containing regimens were associated with reduced frequencies in B-cell subpopulations, whereas lenalidomide-based treatments better preserve the total number of B-cells, highlighting distinct immunological fingerprints associated with each therapy. These effects on cell activation, exhaustion, and isotype switching might present clinical implications for infection risk, vaccine responses, and long-term humoral immunity.^70^ Therefore, these therapies can induce profound immune dysregulation through cell depletion, exhaustion, altered tumor microenvironment, and incomplete immune reconstitution.^15,71–74^

These findings underscore the value of integrative multiomics approaches to disentangle the relative contributions of cellular and humoral immunity to global immune status in oncohematological patients. The predominance of B-cell immunophenotypic features as the principal driver of variance suggested that alterations within the cellular compartment may represent a more sensitive indicator of immune perturbation than humoral immune profiles. While antigen-specific IgM and IgG responses contributed substantially to the integrated model, they explained less heterogeneity, reinforcing that antibody titers alone may not fully capture immune heterogeneity during therapy. From a translational perspective, the strong discriminative performance observed—particularly associated with lenalidomide—suggests distinct therapy-associated immune patterns. Therefore, integrated immune signatures could serve as potential biomarkers of treatment exposure requiring further validation.

The integration of serological signatures with B-cell immunophenotypic profiles presents prognostic potential and could provide novel insights into the biology of the diseases; however, our study is limited in several aspects. The cohort was relatively small, predominantly composed of CLL patients, and treatment groups were closely associated with specific disease entities. Furthermore, the lack of age- and sex-matched healthy controls, the cross-sectional study design, the absence of functional immune assays, and the lack of external validation of the ROC analyses limited the transferability of the results. Despite these limitations, the comprehensive serological and immunophenotypic characterization presented here offers valuable insights into immune dysregulation following the treatment. Future longitudinal studies in larger and more diverse cohorts, complemented by functional immune analyses, will be essential to validate these observations, define the dynamics of immune recovery over time, and determine their clinical outcomes. Importantly, to our knowledge, this is the first study to provide an integrated framework combining high-dimensional serological profiling with detailed B-cell immunophenotyping in a clinically relevant cohort of patients with B-cell hematological malignancies. This multidimensional approach establishes a foundation for future biomarker discovery and precision immune monitoring, aimed at improving patient stratification and therapeutic decision-making.

## 6 Conclusions

Immune dysfunction is a hallmark of oncohematological diseases, shaped by both the underlying disease and treatments such as ibrutinib- and rituximab-containing regimens. Evaluation of pathogen-specific antibody profiles and B-cell immunophenotypes may provide valuable information for immune monitoring and warrants further investigation in larger prospective cohorts. These approaches could support personalized clinical strategies, including vaccination before immunosuppressive treatment, monitoring pathogen-specific IgM and IgG levels, and considering alternative therapies to mitigate the infectious risk.

This study offers an initial immune fingerprint of oncohematological patients across different treatment regimens and highlights the importance of comprehensive immune assessment at diagnosis and prior to therapy. Such an evaluation may enable risk-adapted, personalized treatment decisions and improved monitoring of infections in immunocompromised patients.

## Supplementary Material













## Data Availability

The GenePix raw numerical output data are provided as an Excel file. All Supporting Information is available in PDF format (figures/images) and Excel format (tables).

## Notes

(ZIP)
Detailed information about the microbial antigens included in the array (XLSX)
Detailed information about the B-cell immunophenotyping antibodies used in the gating strategy (XLSX)
Clinical characteristics of the patients (XLSX)
Flow cytometry data for all analyzed cell populations (XLSX)
Flow cytometry gating strategy for the identification of B-cell populations, removal of doublets and dead cells, exclusion of cells not belonging to the B-cell lineage, the strategy employed for identification of the different B-cell populations, quality control immunocompetence protein microarray, overall raw signal fluorescence and log2 (*Z* score) density distribution at each microarray employed, PCA to discard any microarray-wise batch effect, overall raw signal fluorescence and log2 (*Z* score) density distribution at each sample studied, PCA to discard any sample-wise batch effect, PCA to discard any treatment-wise batch effect, PCA of patients according to mAgs distribution along PC1 and PC2, boxplot of the humoral profile IgG against mAgs, median (horizontal line) and interquartile range (box), volcano plots displaying the statistical significance of *Z*-score ratios (*X*-axis) in IgM profile, boxplot of the humoral profile IgM against mAgs, boxplot showing the median (horizontal line) and interquartile range (box), boxplot of the humoral profile against mAgs for the CLL patients, boxplot showing the median (horizontal line) and interquartile range (box), PCA biplot showing the distribution of samples according to B-cell subpopulations and treatment, heatmap showing direct correlation coefficients (simple linear regression) between pathogen-specific antibody responses and B-cell populations stratified by isotype, Global Weights Heatmap, permutation test evaluating the robustness of the ROC curve for predicting clinical response to lenalidomide, and histogram showing the distribution of AUC values obtained from 100 permutations of the outcome labels under the null hypothesis of no association (PDF)

## Abbreviations

AUC: area under the curve
BSA: bovine serum albumin
BTK: Bruton’s tyrosine kinase
CLL: chronic lymphocytic leukemia
DMSO: dimethyl sulfoxide
DN: double-negative B-cells
FACS: fluorescence-activated cell sorting
FBS: fetal bovine serum
FL: follicular lymphoma
FSC: forward scatter
LF: latent factor
mAgs: microbial antigens
MBC: memory B-cells
MM: multiple myeloma
MOFA: multiomics factor analysis
NHL: non-Hodgkin lymphoma
PB: plasmablasts
PBMCs: peripheral blood mononuclear cells
PBS: phosphate buffered saline
PCA: principal component analysis
pre-GC: pregerminal center B-cells
ROC: receiver operating characteristic
RT: room temperature
SSC: side scatter
