# Supplementary material for: Integrated Immune Profiling Identifies Treatment-Associated Immune Patterns in Patients with B‑Cell Hematological Malignancies
Source: J Proteome Res. 2026 Sep 3;25(10):5404–17. doi: 10.1021/acs.jproteome.6c00394 (PMC13640515; doi:10.1021/acs.jproteome.6c00394)
Supplement: Supplementary file 1 [file pr6c00394_si_006.pdf]

Integrated immune profiling identifies treatment-associated immune patterns in patients with B-cell hematological malignancies

Carlota Arias-Hidalgo[1,2,3], Juan Hernández-Vazquez[4], Cristina Teodosio[1,2], Pablo Juanes-Velasco[1,2,3,5], Ana Nuño-Soriano[1,2,3], Paloma Cal-Sabater[4], Elisa Arribas-Rodríguez[4], Aida Fiz-López[4], Marina Pérez-Mazzali[4], Álvaro Martín-Muñoz[6], Pablo Tellería[7], Marina G. De Vicuña[7], Javier Gamazo-Herrero[7], Ignacio de la Fuente-Graciani[8], Sonia Pérez-González[8], Sara Bravo[8], Cristina García-Alonso[8], Carlos Dueñas[7], María Jesús Peñarrubia[8], Manuel Vigo-Aceves[2], Claudia Núñez-Abascal[1], Lucía Neira-González[1], Rafael Gongora[1,2], Sara Cuesta-Sancho[5,9], David Bernardo[5,9,10]*, Ángela-Patricia Hernández[1,2,3,11]*, Manuel Fuentes[1,2,3,5] *.*

* Corresponding author (sara.cuesta.uva@gmail.com, d.bernardo.ordiz@gmail.com, angytahg@usal.es, mfuentes@usal.es)

1. Translational and Clinical Research Program, Cancer Research Center (IBMCC, CSIC-University of Salamanca), Cytometry Service, NUCLEUS, Department of Medicine, University of Salamanca (Universidad de Salamanca), 37008 Salamanca, Spain
2. Instituto de Investigación Biomédica de Salamanca (IBSAL), Complejo Asistencial de Salamanca, Paseo de San Vicente, 58, 37008, Salamanca, Spain
3. Plataforma de Proteómica Clínica, Instituto de Investigación Biomédica de Salamanca, (IBSAL), 37007, Salamanca, Spain
4. Mucosal Immunology Lab. Institute of Biomedicine and Molecular Genetics (IBGM, University of Valladolid-CSIC), 47003, Valladolid, Spain
5. Biomedical Research Networking Centre Consortium of Oncology (CIBERONC), Instituto de Salud Carlos III, 28029, Madrid, Spain
6. Flow Cytometry Facility. Institute of Biomedicine and Molecular Genetics (IBGM, University of Valladolid-CSIC), 47003, Valladolid, Spain
7. Internal Medicine Department, Hospital Clínico Universitario de Valladolid, 47003, Valladolid, Spain
8. Department of Hematology, Hospital Clínico Universitario de Valladolid, 47003, Valladolid, Spain.
9. Instituto de Investigación Biosanitaria de Valladolid (IBioVall), 47010, Valladolid, Spain
10. Centro de Investigaciones Biomédicas en Red de Enfermedades Infecciosas (CIBERINFEC), 28029, Madrid, Spain
11. Department of Pharmaceutical Sciences, Organic Chemistry, Faculty of Pharmacy, University of Salamanca, CIETUS, IBSAL, 37007 Salamanca, Spain

LIST OF SUPPLEMENTARY COMPONENTS

Supplementary Figure 1: Flow cytometry gating strategy for the identification of B cell populations based on acquisition time and FSC/SSC parameters.

Supplementary Figure 2: Quality control immunocompetence protein microarray.

Supplementary Figure 3: Principal Component Analysis (PCA) to discard any treatment-wise batch effect.

Supplementary Figure 4: Principal Component Analysis (PCA) of patients according to mAgs distribution along PC1 and PC2.

Supplementary Figure 5: Boxplot of the humoral profile IgG against mAgs.

Supplementary Figure 6: Volcano plots display the statistical significance of Z-score ratios (X-axis) in IgM profile.

Supplementary Figure 7: Boxplot of the humoral profile IgM against mAgs.

Supplementary Figure 8: Boxplot of the humoral profile against mAgs for the CLL patients.

Supplementary Figure 9: Principal Component Analysis (PCA) biplot showing the distribution of samples according to B-cell subpopulations and treatment.

Supplementary Figure 10: Heatmap showing direct correlation coefficients (simple linear regression) between pathogen-specific antibody responses and B cell populations stratified by isotype.

Supplementary Figure 11: Global Weights Heatmap.

Supplementary Figure 12: Permutation test evaluating the robustness of the ROC curve for predicting clinical response to lenalidomide.

Supplementary Table 1: Detailed information about the microbial antigens included in the array (XLSX file).

Supplementary Table 2: Detailed information about the B cell immunophenotyping antibodies used in the gating strategy (XLSX file).

Supplementary Table 3: Clinical characteristics of the patients (XLSX file).

Supplementary Table 4: Flow cytometry data for all analyzed cell populations (XLSX file).

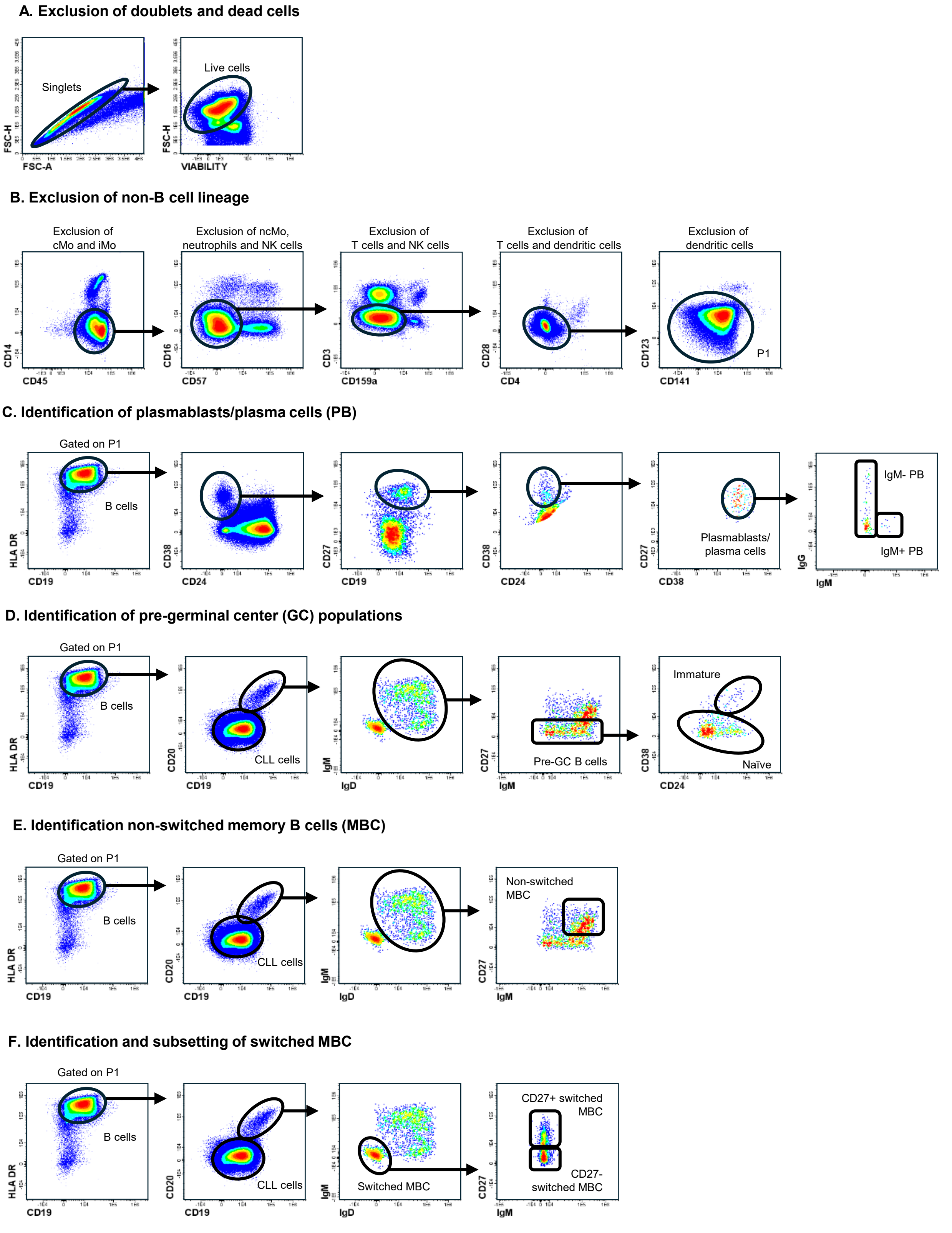

Supplementary Figure 1: Flow cytometry gating strategy for the identification of B cell populations. Cells were initially gated based on acquisition time and FSC/SSC parameters to select PBMCs. Each panel represents a sequential step in the gating strategy. Removal of doublets and dead cells is shown in panel A. Panel B depicts the exclusión of cells not belonging to the B cell lineage. Panels C-F show the strategy employed for identification of the different B cell populations. CLL, chronic lymphocytic leukemia; cMo, classical monocytes; GC, germinal center; iMo, intermediate monocytes; MBC, memory B cells; ncMo, non-classical monocytes; PB, plasmablasts.

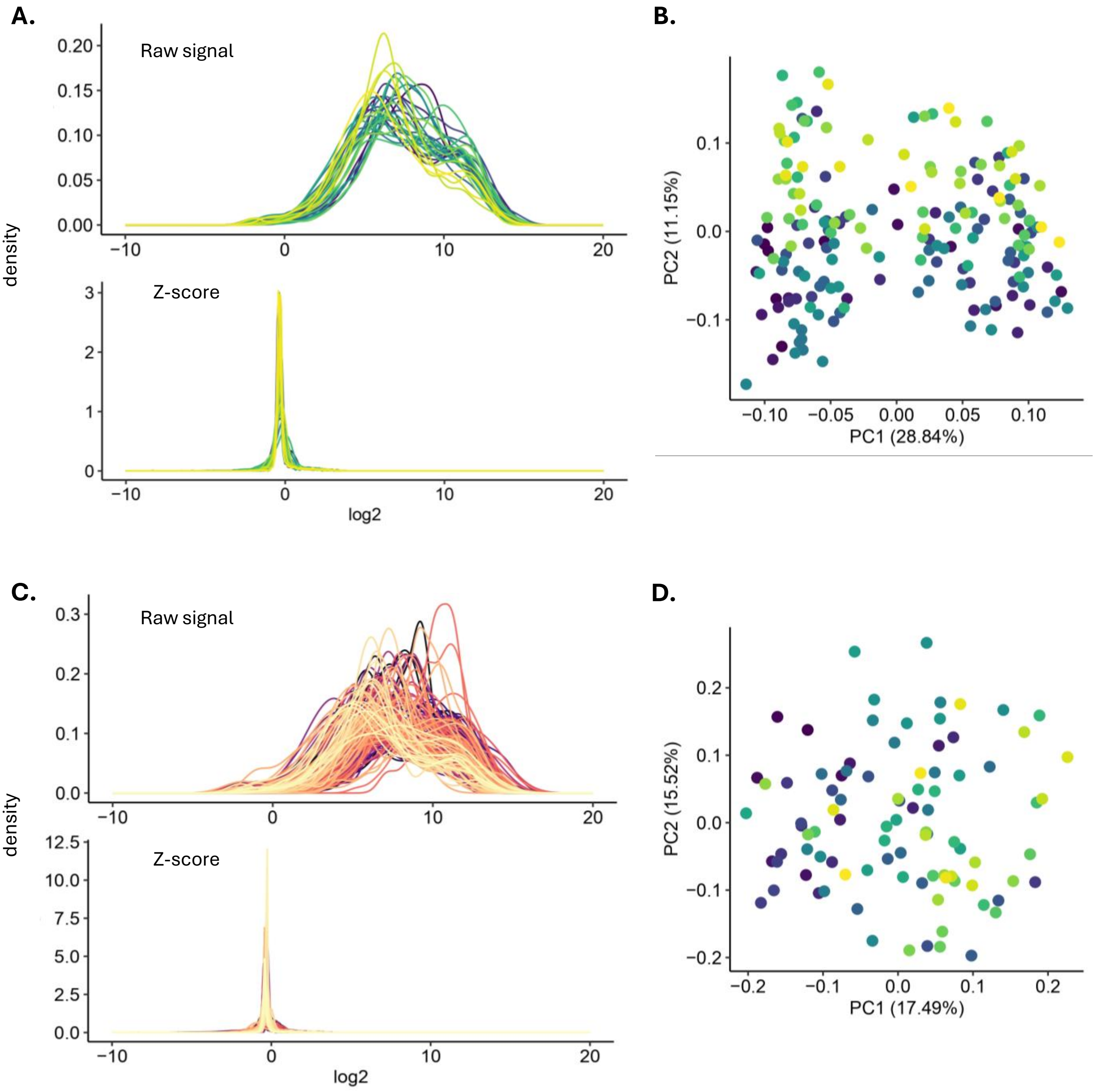

Supplementary Figure 2: Quality control immunocompetence protein microarray. A. Overall raw signal fluorescence and log2 (Z score) density distribution at each microarray employed. B. Principal Component Analysis (PCA) to discard any microarray-wise batch effect. C. Overall raw signal fluorescence and log2 (Z score) density distribution at each sample studied. D. Principal Component Analysis (PCA) to discard any sample-wise batch effect.

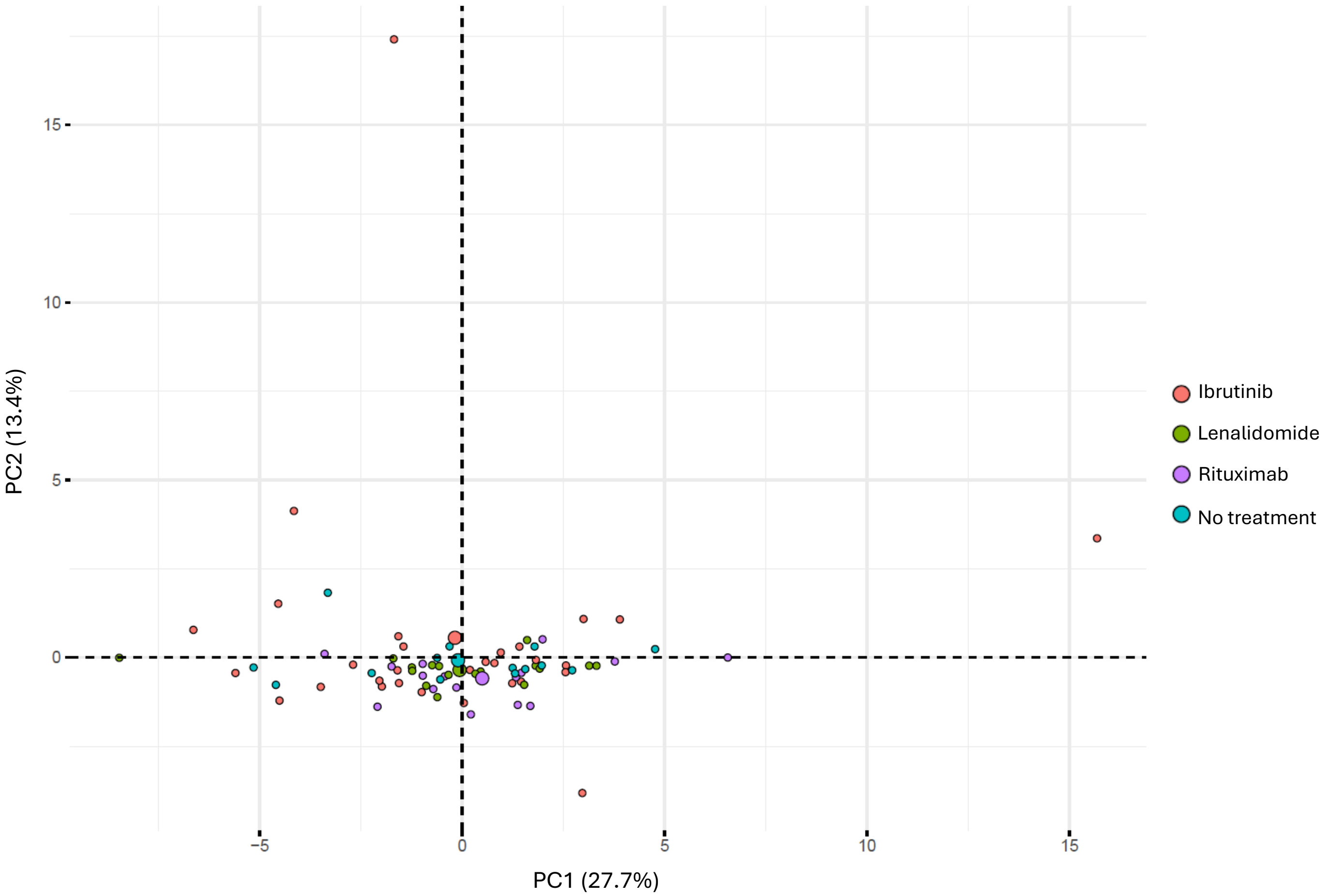

Supplementary Figure 3: Principal Component Analysis (PCA) to discard any treatment-wise batch effect.

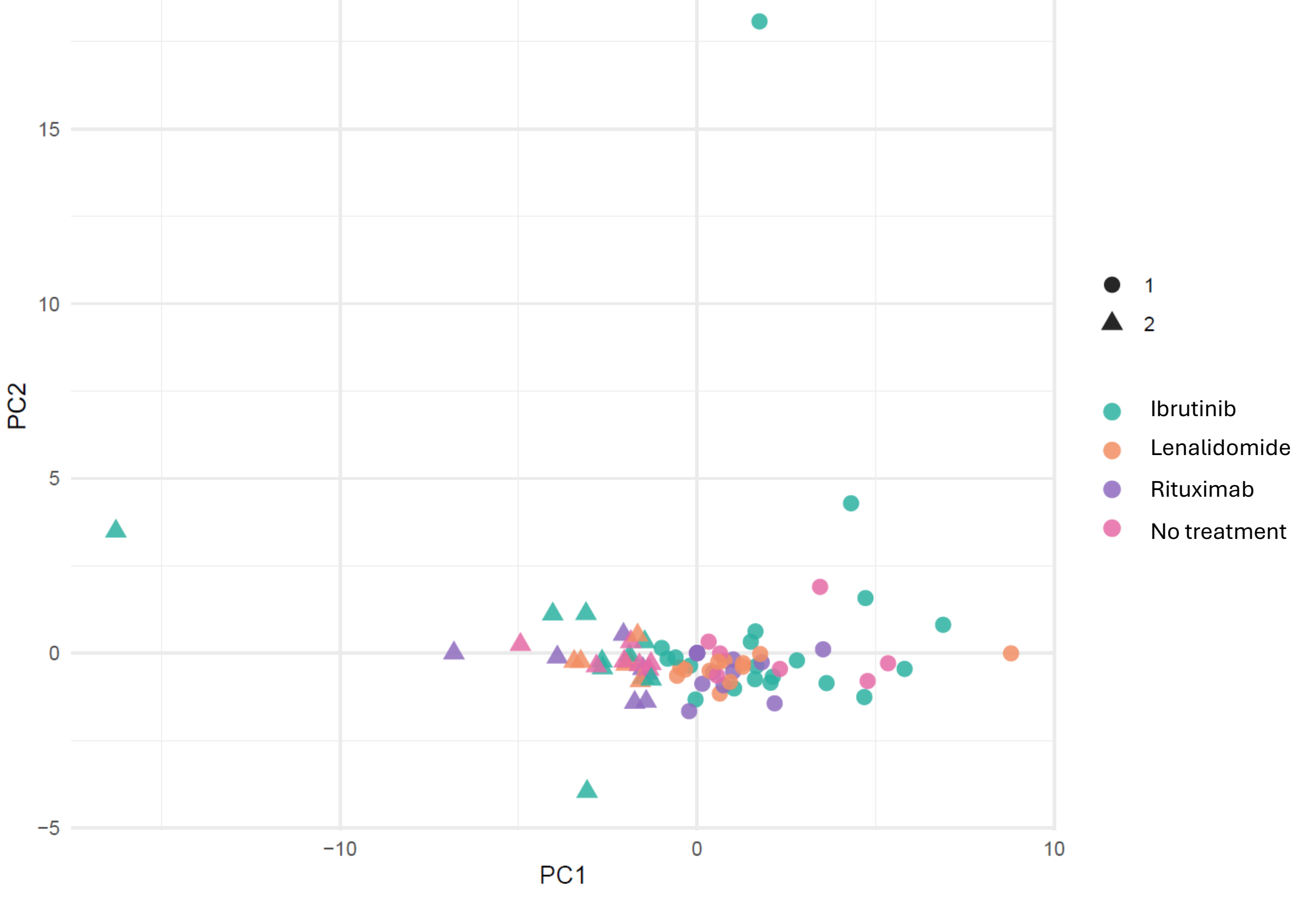

Supplementary Figure 4: Principal Component Analysis (PCA) of patients according to mAgs distribution along PC1 and PC2. Each point represents one patient; colors indicate treatment groups (Ibrutinib, Lenalidomide, Rituximab, and No treatment) and shapes denote cluster assignment.

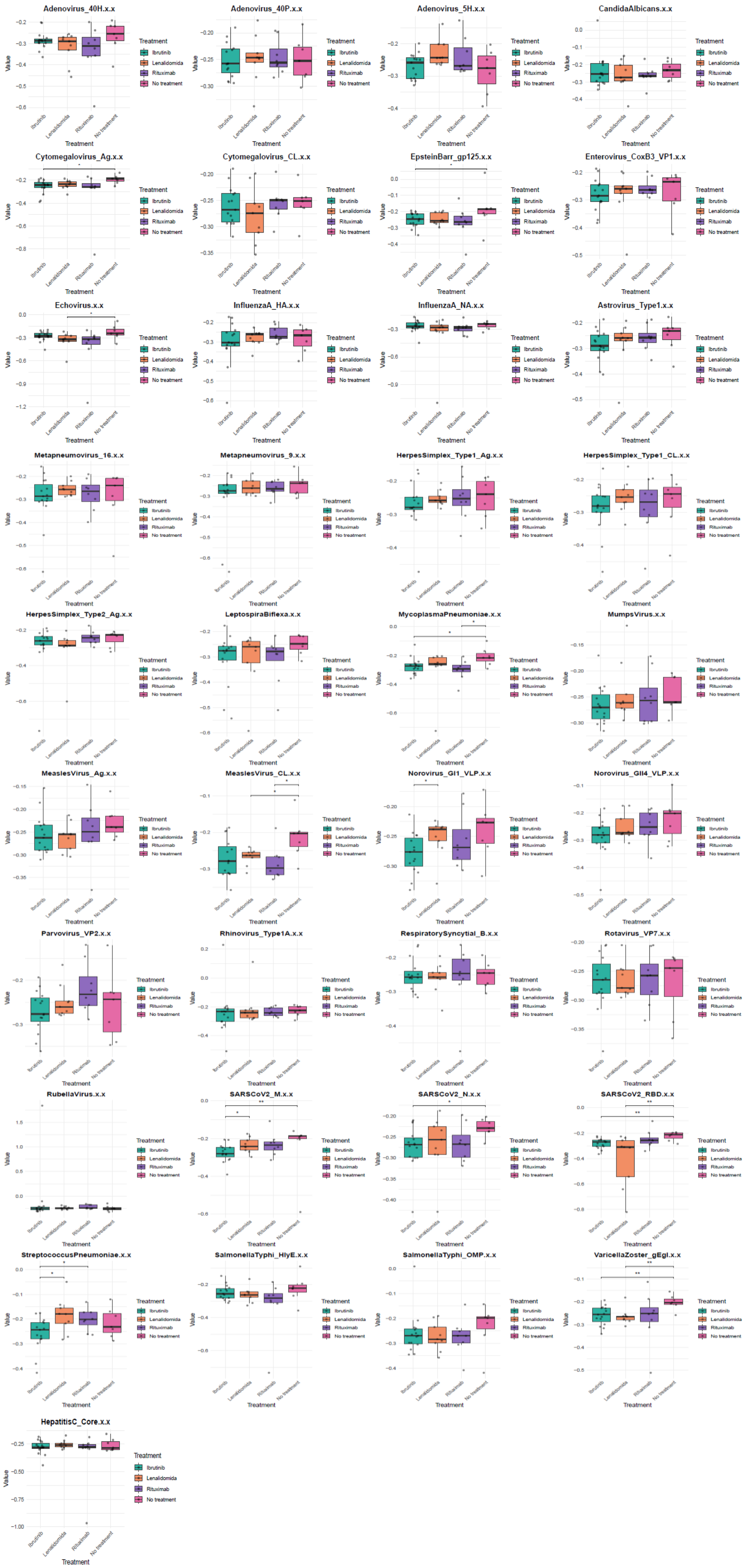

Supplementary Figure 5: Boxplot of the humoral profile IgG against mAgs. The boxplot shows the median (horizontal line) and interquartile range (box). Each color represents a different treatment: green for Ibrutinib, orange for Lenalidomide, purple for Rituximab, and pink for no treatment. Differences between means were performed using the Wilcoxon rank-sum test (p-value: * < 0.05; ** < 0.01).

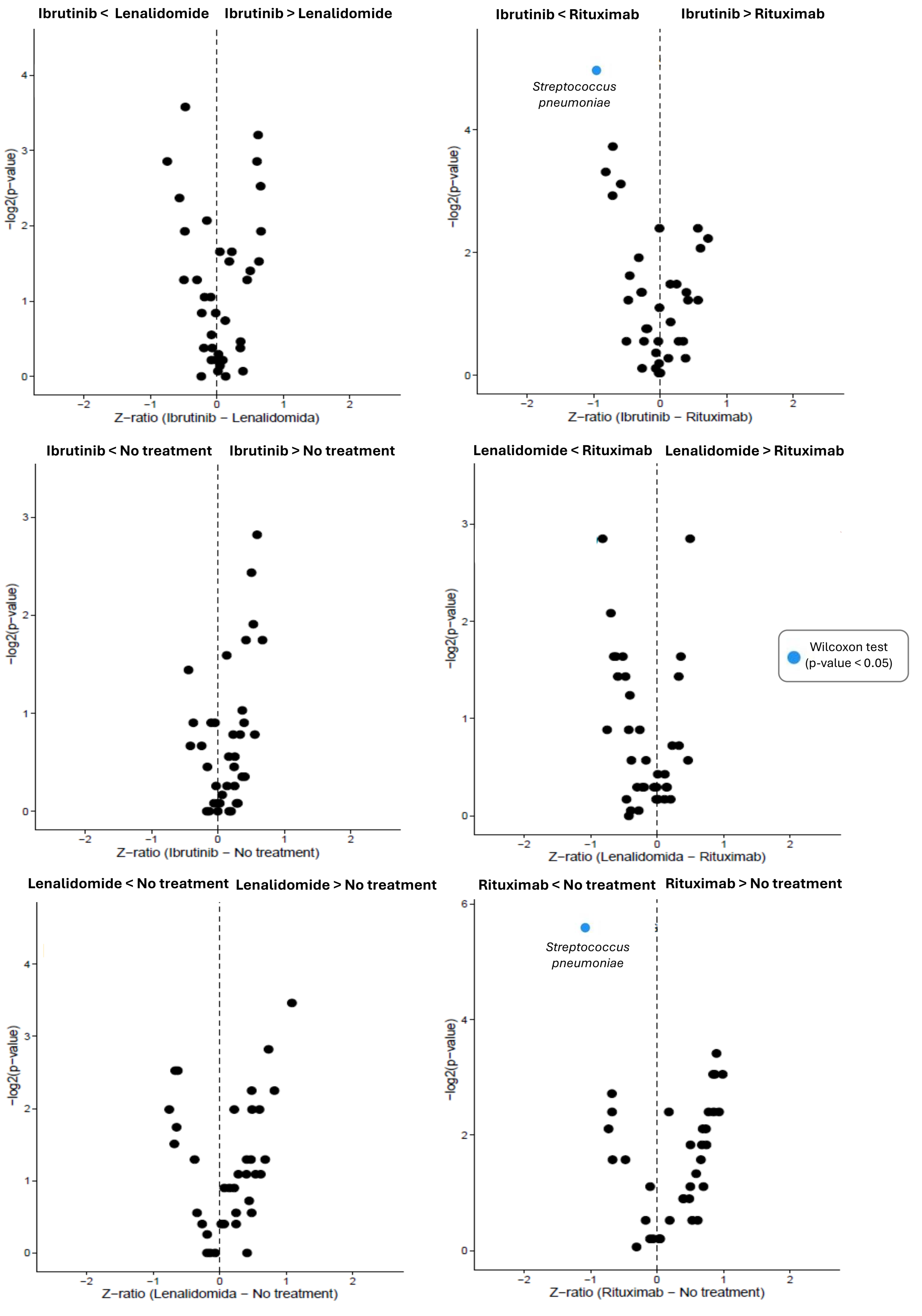

Supplementary Figure 6: Volcano plots display the statistical significance of Z-score ratios (X-axis) in IgM profile. The difference between means was evaluated applying Wilcoxon Rank Sum test (Y-axis). The mAgs with statistically significant differences are highlighted in blue dots. Each volcano plot represents the comparison between the different treatment groups.

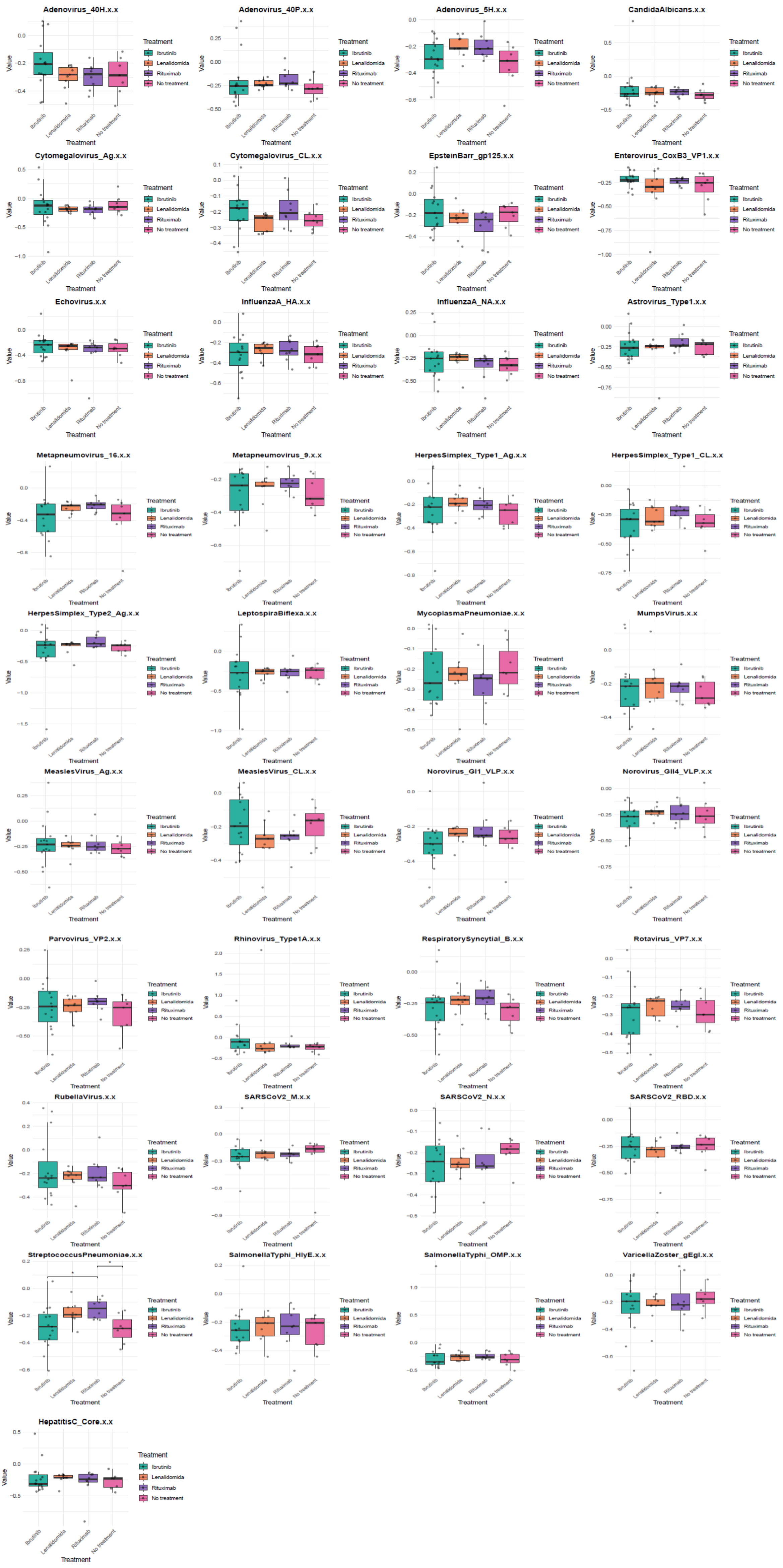

Supplementary Figure 7: Boxplot of the humoral profile IgM against mAgs. The boxplot shows the median (horizontal line) and interquartile range (box). Each color represents a different treatment: green for Ibrutinib, orange for Lenalidomide, purple for Rituximab, and pink for no treatment. Differences between means were performed using the Wilcoxon rank-sum test (p-value: * < 0.05).

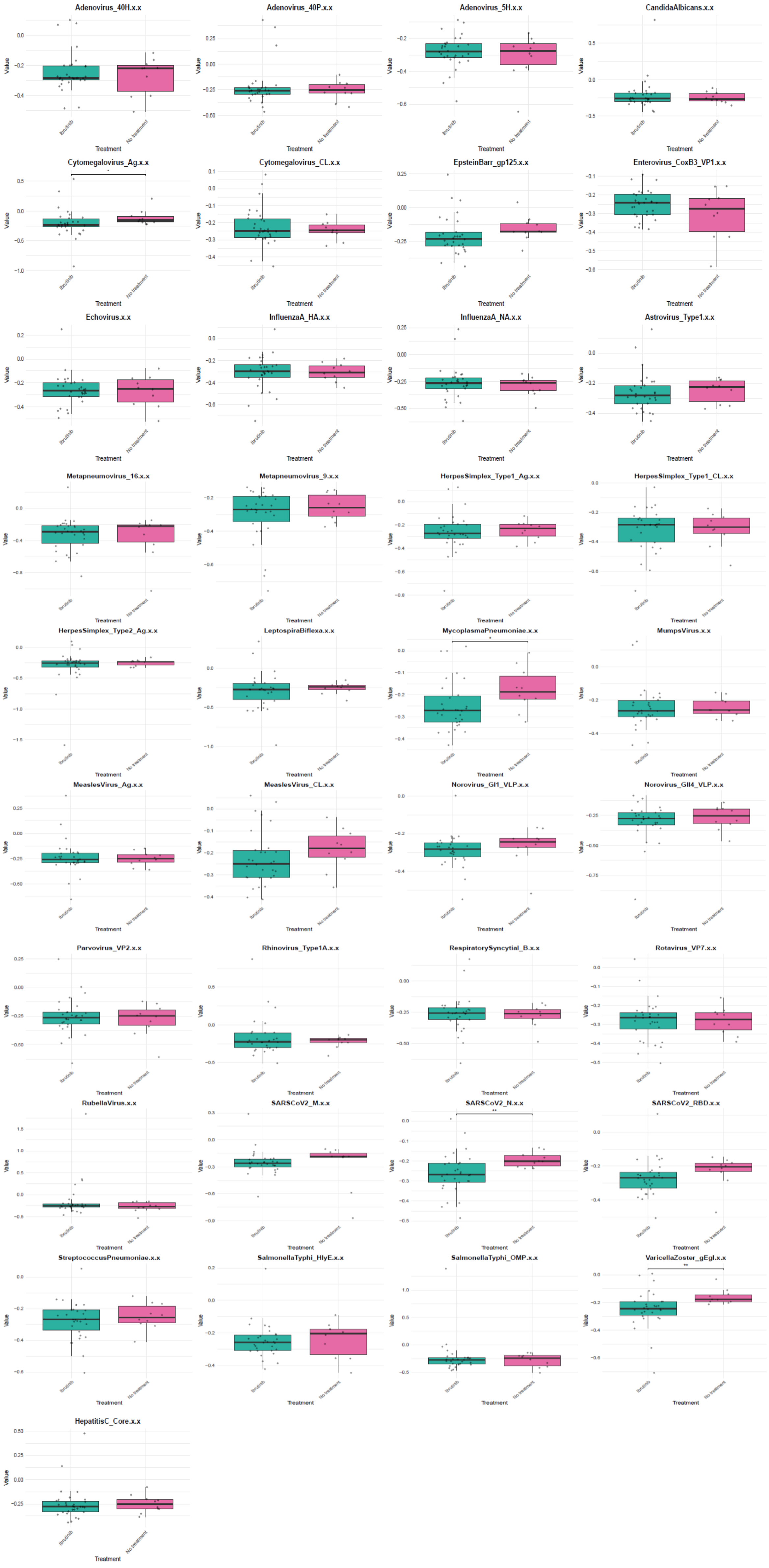

Supplementary Figure 8: Boxplot of the humoral profile against mAgs for the CLL patients. The boxplot shows the median (horizontal line) and interquartile range (box). Green represents the Ibrutinib-treated group, and pink represents the non-treated group. Differences between means were performed using the Wilcoxon rank-sum test (p-value: * < 0.05; ** < 0.01).

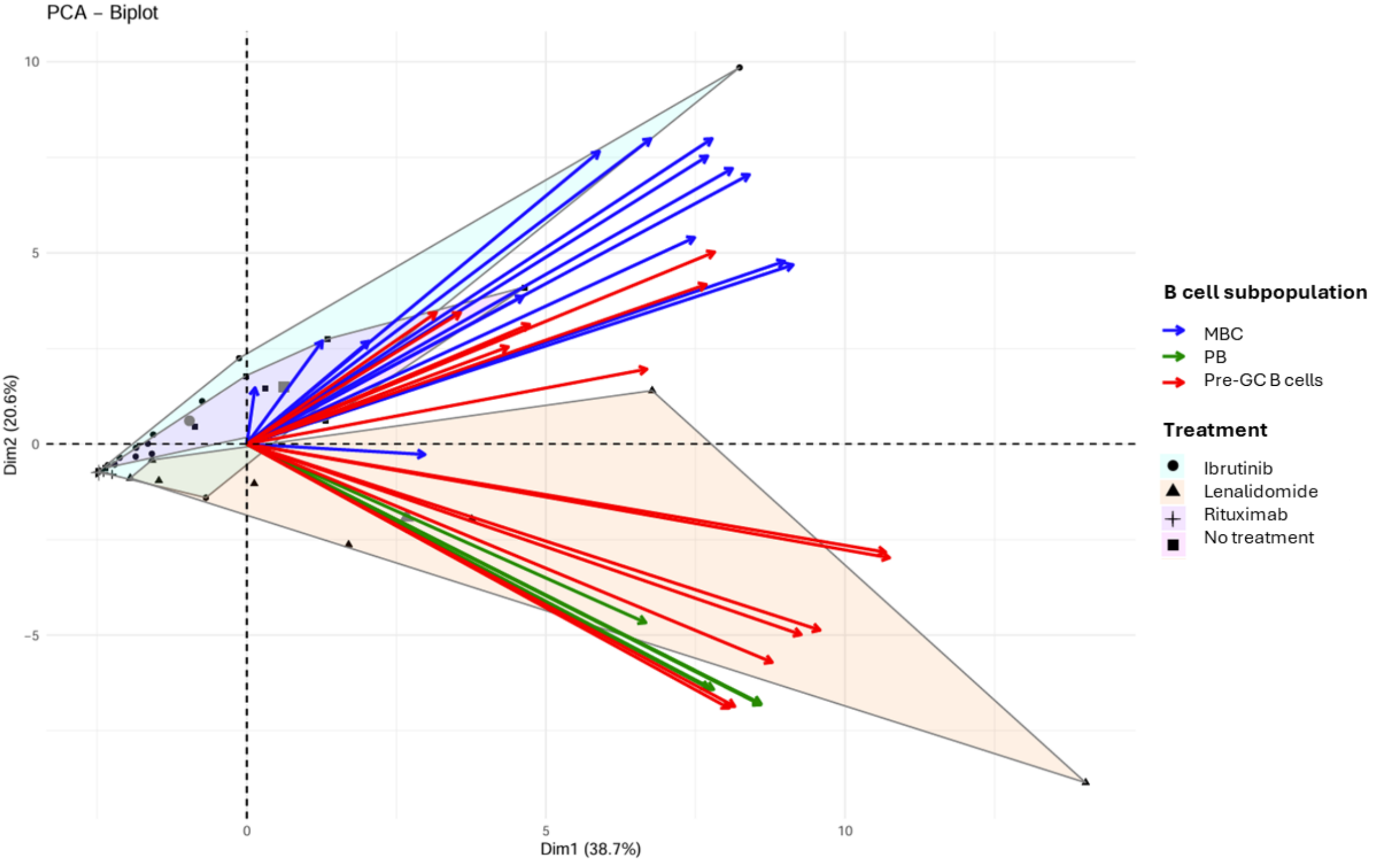

Supplementary Figure 9: Principal Component Analysis (PCA) biplot showing the distribution of samples according to B-cell subpopulations and treatment. Arrows represent the contribution and direction of B-cell subpopulations (memory B cells, MBC; plasmablasts, PB; and pre-germinal center B cells, Pre-GC) to the first two principal components. Points correspond to individual samples, colored and shaped according to treatment (Ibrutinib, Lenalidomide, Rituximab, or no treatment). Shaded polygons indicate the dispersion of samples within each treatment group. Dim1 and Dim2 explain 38.7% and 20.6% of the total variance, respectively.

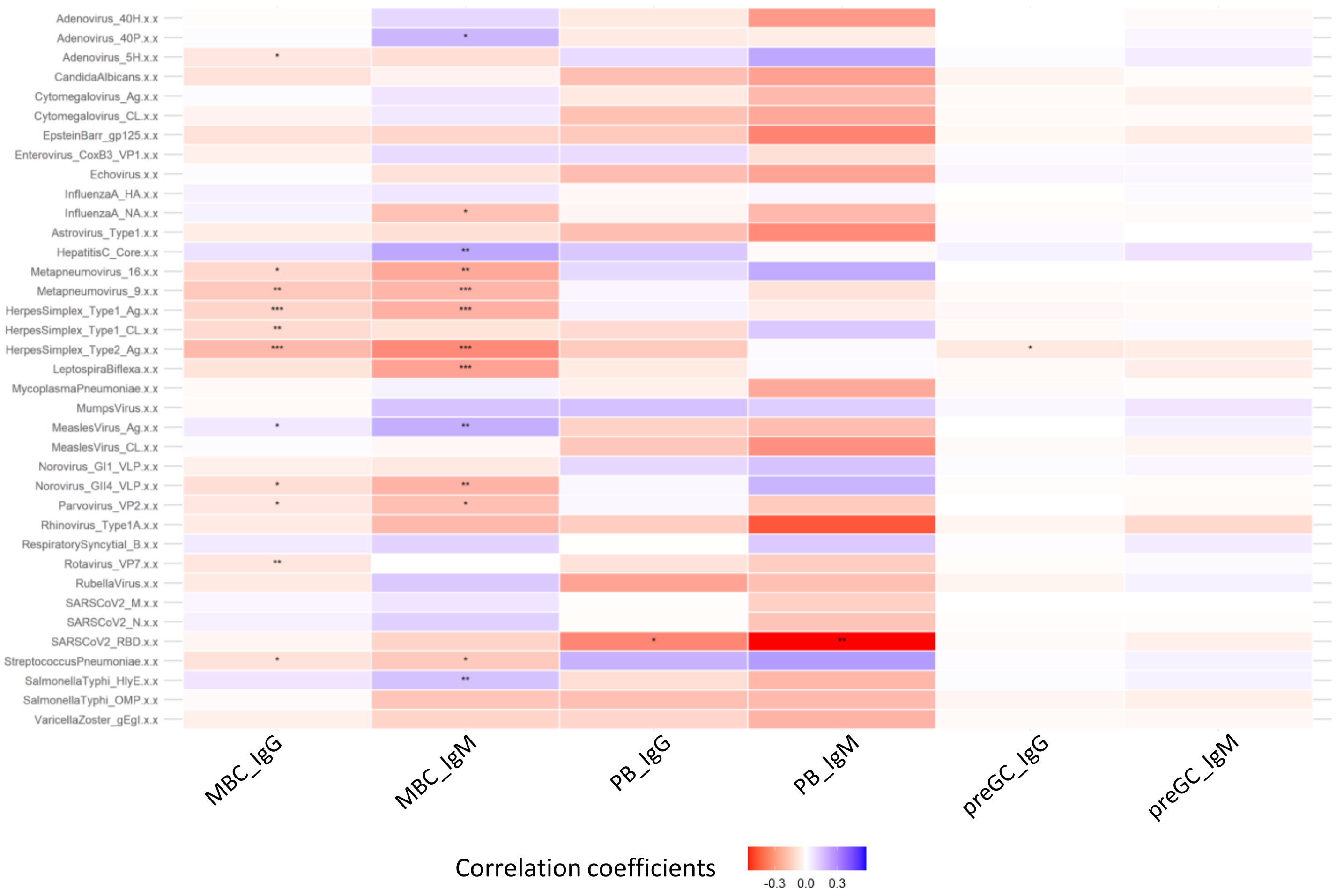

Supplementary Figure 10: Heatmap showing direct correlation coefficients (simple linear regression) between pathogen-specific antibody responses and B cell populations stratified by isotype. Rows represent individual pathogens, and columns correspond to memory B cells (MBC), plasmablasts (PB), and pre–germinal center (preGC) B cells, each separated by IgG and IgM responses. Blue indicates positive correlations, whereas red denotes negative correlations. Color intensity reflects the magnitude of the correlation coefficient. Asterisks indicate statistically significant associations (*p < 0.05; **p < 0.01; ***p < 0.001), with multiple significant correlations observed particularly within the MBC compartment.

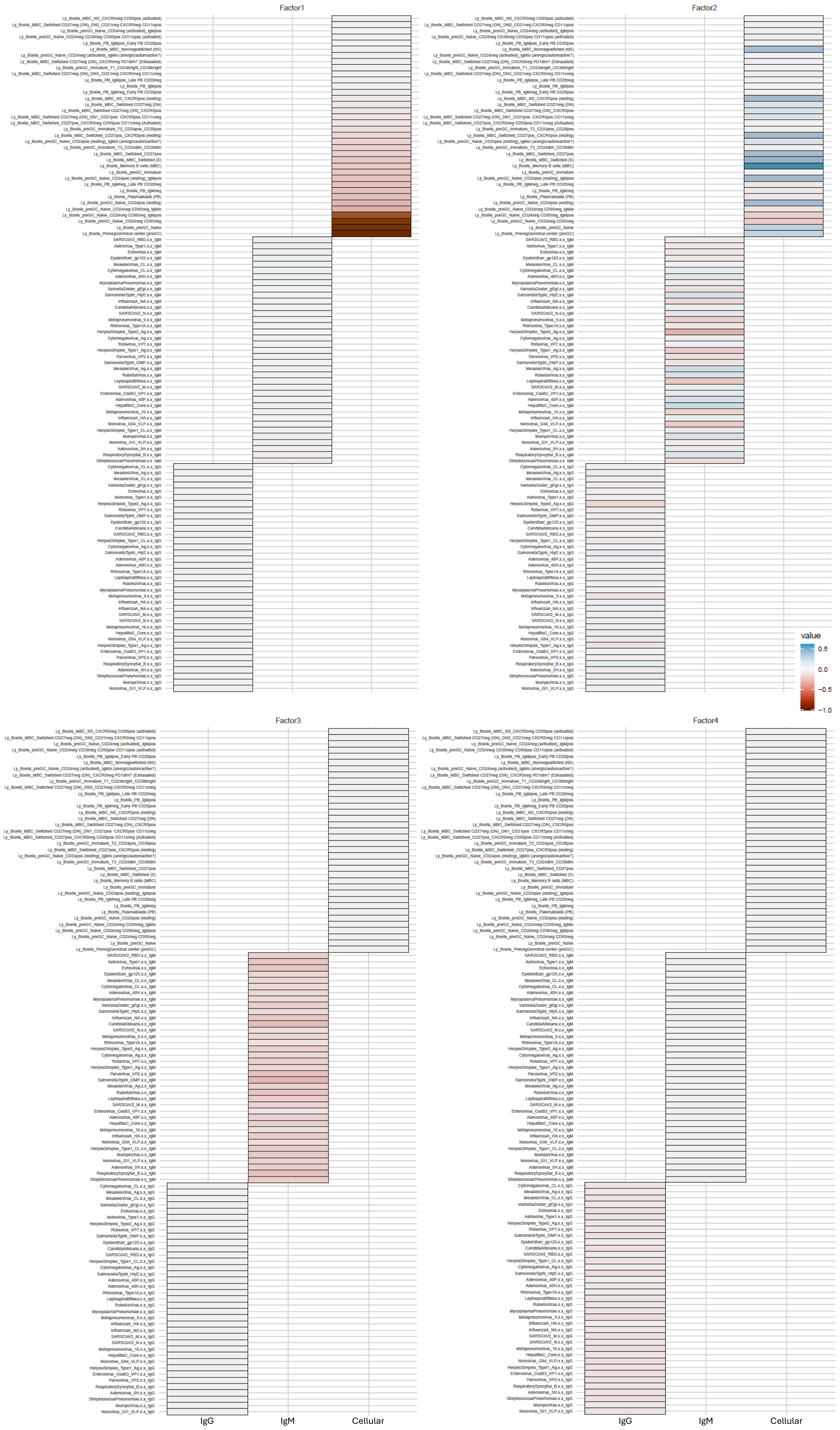

Supplementary Figure 11: Global Weights Heatmap.

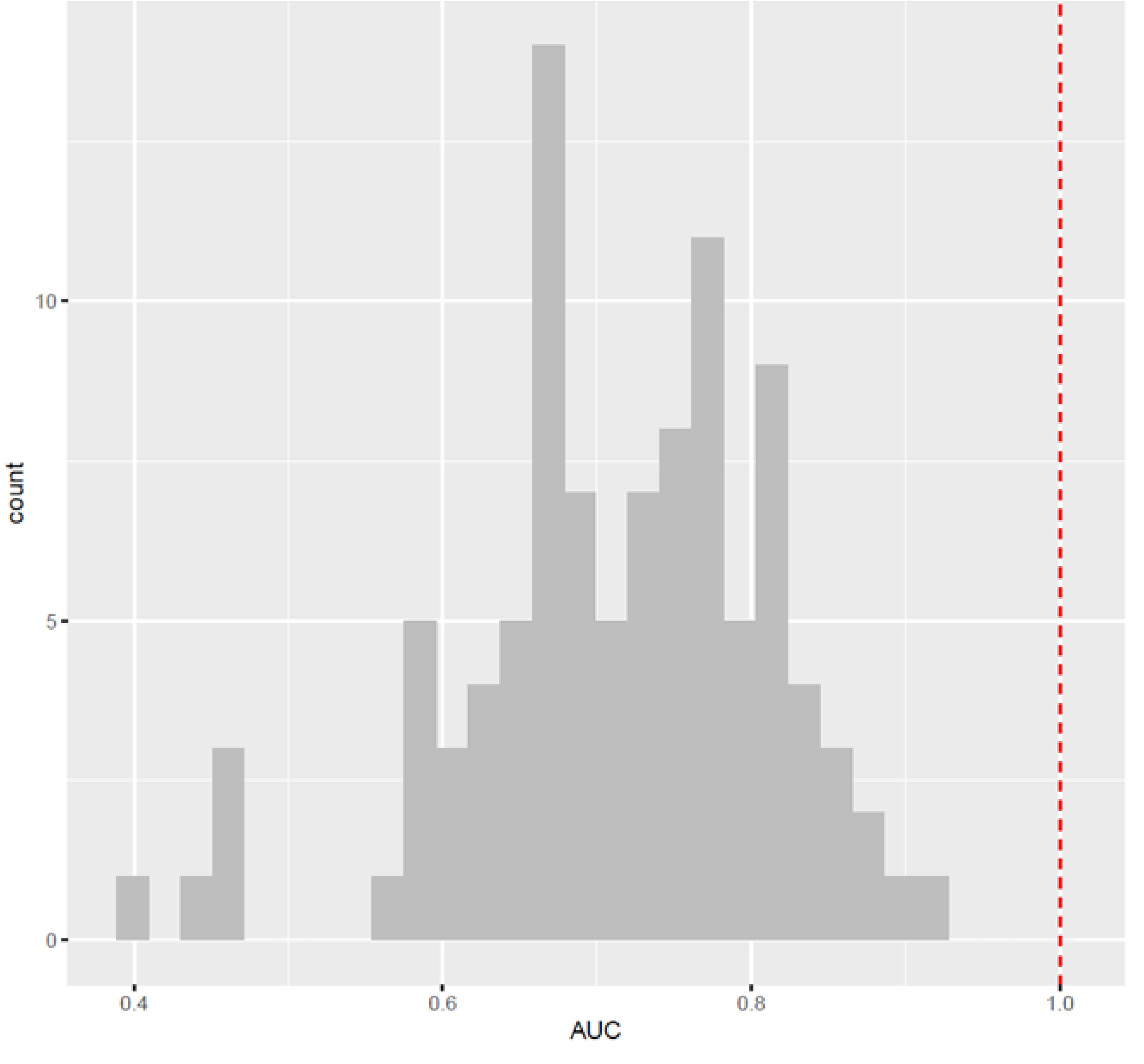

Supplementary Figure 12: Permutation test evaluating the robustness of the ROC curve for predicting clinical response to lenalidomide. The histogram shows the distribution of AUC values obtained from 100 permutations of the outcome labels under the null hypothesis of no association. The red dashed line indicates the real AUC (1.0).
